# Pathways and products of base excision DNA repair in *Xenopus laevis* eggs: contrast with human cell pathways

**DOI:** 10.1093/nar/gkaf1326

**Published:** 2025-12-10

**Authors:** Zhouyiyuan Xue, Patrick James Sutton, John D Haley, Christopher W Brownlee, Bruce Demple

**Affiliations:** Department of Pharmacological Sciences, Renaissance School of Medicine, Stony Brook University, Stony Brook, NY 11794, United States; Program in Molecular and Cellular Biology, Stony Brook University, Stony Brook, NY 11794, United States; Department of Pharmacological Sciences, Renaissance School of Medicine, Stony Brook University, Stony Brook, NY 11794, United States; Department of Pathology, Renaissance School of Medicine, Stony Brook University, Stony Brook, NY 11794, United States; Department of Pharmacological Sciences, Renaissance School of Medicine, Stony Brook University, Stony Brook, NY 11794, United States; Department of Pharmacological Sciences, Renaissance School of Medicine, Stony Brook University, Stony Brook, NY 11794, United States

## Abstract

Endogenous DNA damage is mutagenic and cytotoxic unless removed by base excision DNA repair (BER). BER replaces 2–10 nucleotides in “long patch” (LP-BER) or just one nucleotide in the simpler “short-patch” SP-BER. LP-BER is required for some oxidative lesions. *Xenopus laevis* egg extracts (HSS) had robust BER of diverse lesions (uracil and various abasic sites). It was surprising to find no evidence for HSS repair of 8-oxoguanine, an important mutagenic lesion. Also unexpected was the *independence* of HSS BER from DNA polymerase β (Polβ), which typically contributes both DNA repair synthesis and 5′ “trimming” to SP-BER. Instead, HSS BER was blocked by aphidicolin inhibiting the replication DNA polymerases δ and ε. Added human Polβ restored full repair, requiring only its 5′-trimming activity, not its DNA polymerase function. Mass-labeled probes revealed that *Xenopus* BER repair patches were 80%–90% 2-nucleotide (2-nt) LP-BER tracts. Supplementing the low Polβ level in HSS with recombinant enzyme shifted BER toward 1-nt (SP-BER) patches, also influenced by the surrounding sequence. Excision *upstream* (5′) of the lesion occurred in most of the frog BER products. In contrast, human cell (HEK293) extracts had Polβ-dependent BER, with mostly 2-nt repair patches. Human LP-BER was driven by the Fen1 flap endonuclease, but it did not include 5′ excision.

## Graphical Abstract

**Graphical Abstract:**
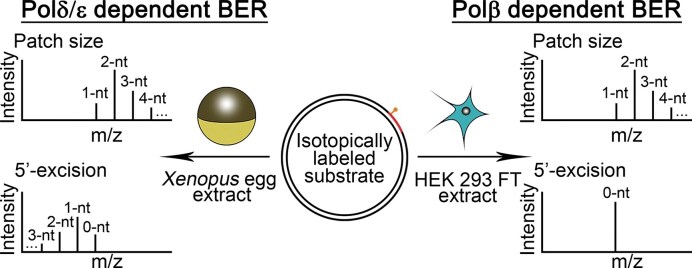

## Introduction

Cellular DNA is damaged by environmental agents, but many DNA lesions also occur endogenously, via incessant hydrolytic reactions such as depurination and base deamination, and reactions with metabolic products such as free radicals and aldehydes [1–3]. The cytotoxic and mutagenic potential of DNA damage is counteracted by DNA repair systems, most notably BER, which constitutes the front-line defense against the bulk of endogenous DNA damage. BER is typically initiated by a DNA glycosylase removing a damaged or inappropriate base, for example by the enzyme uracil-DNA glycosylase (UDG) [4, 5]. This reaction produces an abasic site like those generated by spontaneous depurination, and these are all incised by an AP endonuclease, such as the Ape1 protein of vertebrates [6]. Incision by Ape1 generates a normal 3′-hydroxyl nucleotide, which is extended during DNA repair synthesis [7]. In vertebrates, Polβ is most prominently associated with BER, and this protein contains a separate domain with deoxyribose-5-phosphate (dRp) lyase activity that can excise the 5′-dRp residue left by Ape1 incision [8]. In this model, the combined insertion of a single nucleotide and removal of the 5′-dRp generates a nicked product, allowing BER to be completed by a DNA ligase [8, 9]. This process is often referred to as SP-BER. However, LP-BER, which involves the replacement of multiple nucleotides, has also been widely reported [10–13]. As a result, to enable DNA ligation, LP-BER requires an additional activity, such as Fen1, to remove the displaced 5′ “flap” [14].

It is uncertain whether and how there is a switch between the SP- and LP-BER pathways, nor which DNA polymerases provide the longer repair patches. This is an important question, because some oxidative DNA lesions absolutely require LP-BER [9], and the expression of DNA polymerases can change dramatically during the cell cycle. For example, the terminal differentiation of muscle cells [15] or neuronal precursor cells [16] is accompanied by dramatic down-regulation of Fen1, which greatly limits LP-BER in the nucleus. With differentiation into myotubes, mouse myoblasts lose significant BER capacity and have increased sensitivity to oxidative DNA damage [15], consistent with a loss of LP-BER capacity. This deficiency likely reflects not only Fen1 down-regulation but also the decreased levels of the replication enzymes DNA polymerase δ (Polδ) and DNA polymerase ε (Polε), which are more likely to generate longer DNA synthesis tracts than the poorly processive Polβ [17, 18].

Studies of BER pathways have depended almost entirely on the use of cell-free extracts, which for mammalian cells are (usually) inherently a mixture representing all stages of the cell cycle. The studies of terminally differentiated cells mentioned earlier [15, 16] represent one extreme in which DNA replication in the nucleus is permanently halted. However, during the typical vertebrate cell cycle, the levels of DNA replication proteins, including those implicated in LP-BER, fluctuate from a low point in G_1_ to a maximum during S-phase [19]. If the replication polymerases are indeed required for LP-BER, then the cellular capacity for this process would be more limited in G_0_/G_1_ than in S-phase. Conversely, SP-BER may be more active in G_0_/G_1_ than in S [2]. *Xenopus* eggs offer a unique opportunity because they are poised for rapid DNA replication immediately following fertilization. Consistent with this situation, the expression levels of Polδ, Polε, and Fen1 proteins are all much higher than that of Polβ. To study the *Xenopus* BER pathways, we improved a mass-labeled BER probe that enables a direct readout of SP- and LP-BER, showing that multinucleotide BER is predominant in the eggs. We compare these results with new data obtained for human cell (HEK293) extracts, which show how the SP- and LP-BER distribution can be influenced by total protein concentration, the surrounding DNA sequence, and the availability of Fen1 protein.

## Materials and methods

### Competent cells and transformation

To prepare DH5α competent cells, DH5α cells (NEB, C2988J) were inoculated into 50 ml of LB broth (0.5% yeast extract, 1% tryptone, and 1% NaCl) and incubated overnight at 37°C and 250 rpm. Subsequently, a 50 ml cell culture was diluted into 1 L of LB broth and incubated for 1.5 h at 37°C and 250 rpm. During this period, all buffers and equipment were pre-cooled in a 4°C cold room, and the subsequent steps were conducted there as well. Afterward, the culture was chilled on ice for 15 min, followed by the collection of DH5α cells through centrifugation at 1500 × *g* for 10 min at 4°C. The resulting cell pellet was then resuspended in 40 ml of washing buffer (0.08 M MgCl_2_ and 0.02 M CaCl_2_). This suspension was chilled on ice for 10 min and subjected to centrifugation at 1500 x *g* for 10 min at 4°C. The resulting pellet was resuspended in the washing buffer again, followed by another round of chilling and centrifugation. Finally, the pellet was resuspended in 2 ml of storage buffer using pipetting, and storage buffer (0.05 M CaCl_2_ and 20% glycerol) was added to bring the volume to 40 ml. The resulting suspension was aliquoted into 1.5 ml Eppendorf tubes, with each tube containing 100 μl, and stored at −80°C.

For the transformation procedure, DH5α competent cells were thawed on ice. Subsequently, 50 ng of sample DNA and 10 ng of pUC19 control DNA (NEB, N3041S) were separately added to the thawed competent cells, and the tubes were flicked gently. After a 30-min incubation on ice, the competent cells were subjected to a 15-s heat shock in a 42°C water bath, followed by an immediate return to ice. The reaction was then supplemented with 800 μl of SOC buffer (0.5% yeast extract, 2% tryptone, 10 mM NaCl, 2.5 mM KCl, 10 mM MgCl_2_, 10mM MgSO_4_, and 20 mM glucose) and incubated at 37°C with shaking at 250 rpm for 1 h. The culture was then diluted at ratios of 1:10, 1:50, and 1:200, with 100 μl of the diluted culture spread onto a 100 μg/ml ampicillin-selective plate (Sigma–Aldrich, A9518). After overnight incubation at 37°C, five single-cell colonies were selected and individually inoculated into 6 ml of LB broth with 100 μg/ml ampicillin (Sigma–Aldrich, A9518). These cultures were incubated at 37°C with shaking at 250 rpm for 16 h. Subsequent plasmid purification was performed using miniprep spin columns in accordance with the manufacturer’s protocol, and the resulting plasmids were sequenced by the Stony Brook University Genomics Core Facility. Five hundred microliters of DH5α cells that carry the correct plasmid were mixed with 500 μl of 50% glycerol and stored at –80°C.

### Oligonucleotides

All of the synthetic oligonucleotides used in this study are listed in Supplementary Table S1, along with their sources.

### Construction of the N3–1 plasmid vector

The process commenced with the expansion of pUC19 (NEB, N3041S). Following the previously described procedure, pUC19 was transformed into DH5α cells. pUC19 plasmid was purified by using miniprep spin columns (Qiagen, 27104). Linearization of pUC19 was achieved using SalI (NEB, R0138S) and BamHI (NEB, R3136S), and the resulting linear fragment was purified through agarose gel electrophoresis and gel extraction using QIAquick gel extraction kit (Qiagen, 28704). A reaction mixture of 30 ng linearized pUC19, 0.2 pmol ssN3-1 V1 X:A (Integrated DNA Technologies), and 10 μl 2× NEBuilder HiFi DNA Assembly Master Mix (NEB, E2621S) was prepared, followed by incubation at 50°C for 1 h. Five microliters of the ligation product was transformed into 100 μl of chemically competent DH5α cells, as previously described. The resulting single-cell colonies were expanded, and the plasmids they carried were purified using the miniprep spin column. Subsequent plasmid sequencing confirmed the sequence. To eliminate an extra Nt.BspQI site located outside the N3 region, HindIII (NEB, R0104S) and AflIII (NEB, R0541S) enzymes were employed. Using 30 units each of HindIII and AflIII, 10 μg of previously purified plasmid was linearized in NEB buffer r2.1 (NEB, B6002S) in a total reaction volume of 50 μl. Following 1 h of incubation at 37°C, heat inactivation of the restriction enzymes was carried out at 80°C for 20 min. For the blunting step, nine units of T4 DNA polymerase (NEB, M0203S), 5 nmol of dNTPs (NEB, N0446S), and 1 μl of 10× NEB buffer r2.1 (NEB, B6002S) were added to the reaction mixture, with H_2_O used to reach a final volume of 60 μl. Blunting was carried out at 12°C for 15 min. The resulting blunted fragment was purified through electrophoresis and gel extraction and then resuspended in 30 μl of H_2_O. Subsequently, a mixture consisting of 1200 units of T4 DNA ligase (NEB, M0202S), 5 μl of 10× T4 DNA ligase buffer (NEB, B0202S), 30 μl of DNA, and H_2_O to a final volume of 50 μl was incubated at 25°C for 4 h for blunt end ligation. A portion of 5 μl of the resulting product was transformed into 100 μl of chemically competent DH5α cells. Plasmids purified from the secondary transformation were subjected to DNA sequencing to confirm the removal of the extra Nt.BspQI site. The final plasmid was named N(3–1) Ver 1 X:A. The plasmids N(3–1) Ver 1 X:G, N(3–1) Ver 6 X:A, and N(3–1) Ver 7 X:A were constructed in the same way, using different single-stranded oligos (Integrated DNA Technologies, Ultramer DNA Oligonucleotides).

### Preparation of biotinylated, mass-labeled BER substrate containing uracil

DH5α cells carrying N3–1 were inoculated into 10 ml of LB broth and incubated at 37°C with shaking at 250 rpm for 8 h. This culture was then added to 2 L of LB broth and further incubated at 37°C with shaking at 250 rpm for 16 h. The N(3–1) Ver 1 X:A plasmid was purified using a Megaprep kit (Qiagen, 12181) and aliquoted into 100 pmol per tube, stored at −20°C. To synthesize the substrate, 100 pmol of N(3–1) Ver 1 X:A was nicked using 120 units of Nt.BspQI (NEB, R0644S) in NEB r3.1 (NEB, B6003S) at 50°C for 1 h in a total reaction volume of 500 μl. Subsequently, 100 units of Nb.BbvCI (NEB, R0631L) were introduced into the reaction mixture and incubated at 37°C for 2 h. The nickases were then heat-inactivated by incubating at 80°C for 20 min.

Following this, 450 pmol of N3–1 U iso G (Yale University School of Medicine, W.M. Keck Oligonucleotide Synthesis Facility) was added to the reaction mixture, and the heat block was slowly cooled down by placing it in a foam box without capping to allow the oligonucleotide swap to take place. The tube was then incubated in another 65°C heat block for 5 min and slowly cooled. Once the reaction mixture cooled to 37°C, 20 μl of 50 mM ATP (Thermo Fisher Scientific, J61125.14), 800 units of T4 DNA ligase, and 10 μl of 500 mM MgCl_2_ were added, along with H_2_O to achieve a final volume of 1000 μl. The ligation was then performed at 37°C for 3 h (or 1 h at 37°C, followed by overnight incubation at 16°C).

To purify the product, 1 ml of phenol-chloroform (Sigma–Aldrich, P2069) was added into the reaction mixture and vortexed for 30 s to denature the proteins. Subsequently, the tube was centrifuged at 17 900 × *g* for 5 min at 4°C. The upper supernatant was transferred to a new centrifuge tube, and 0.1 volume of 3 M sodium acetate and 0.7 total volume of isopropanol were added to precipitate plasmid. This mixture was placed at −20°C for 1 h and then centrifuged at 17 900 × *g* for 30 min at 4°C. The supernatant was discarded, and the pellet was washed with 70% ethanol at room temperature, followed by centrifugation at 17 900 × *g* for 5 min at 4°C. The pellet was air-dried and subsequently resuspended in 40 μl of H_2_O.

Then, 20 units of exonuclease I (NEB, M0293S), 50 units of exonuclease III (NEB, M0206S), 5 μl of 10× NEB buffer 1 (NEB, B7001S), and 3.5 μl of H_2_O were introduced into the sample to remove any excess single-strand oligos and substrates containing nicks or AP sites. This cleaning step was carried out at 37°C for 1 h in a total reaction volume of 50 μl. Subsequently, phenol-chloroform (Sigma–Aldrich, P2069) was utilized to eliminate all proteins, and isopropanol was employed to precipitate the substrate, following the procedure described earlier. The N(3–1) U:A substrate with mass-labeled G residues was resuspended in 50 μl of H_2_O, and its concentration was assessed using a NanoDrop Spectrophotometer.

To synthesize N3–1 V6 or N3–1 V7 plasmid substrate, 1200 pmol enzymatically labeled oligo was added to 100 pmol nicked plasmid backbone and followed the same procedure as earlier.

A reaction mixture was prepared to assess the substrate’s purity by combining 1 μg of the Bio-T U mass-labeled G BER substrate, 2.5 units of UDG (NEB, M0280S), and 2.5 μl of 10× rCutSmart buffer (NEB, B6004S) in a 25 μl volume. After a 30-min incubation at 37°C, 20 units of NdeI (NEB, R0111S) were added to the reaction, followed by an additional 1-h incubation at 37°C. A separate set of reactions was conducted to create markers for this reaction. In these reactions, 1 μg of the U:A substrate with mass-labeled G residues was digested using 20 units of NdeI (NEB, R0111S) or 20 units of PstI (NEB, R0140S) in rCutSmart buffer, each in a total reaction volume of 25 μl. All three reactions, including the main reaction and the control groups, were quenched by adding 5 μl of 6× SDS gel loading dye (NEB, B7024S). The resulting mixtures were then resolved on an ethidium bromide-containing 0.8% agarose gel. The digestion product from NdeI (NEB, R0111S) served as a marker for plasmids that failed in the oligo-swapping process, while the BamHI (NEB, R0136S) digestion product served as a marker for plasmids that successfully underwent the oligo-swapping process.

### Preparation of negatively supercoiled plasmid substrates

Purified plasmid substrate (150 μg at ∼1.5 μg/μl concentration) was mixed with 0.5 μl Supercoil-It HS enzyme (Bayou Biolabs, S-101), 20× Supercoil-It buffer (Bayou Biolabs, S-101), and H_2_O to achieve 1× final buffer concentration. The mixture was incubated at 37°C for 3 h. Enzymes were removed through phenol-chloroform extraction, and the resulting supercoiled plasmid was precipitated using isopropanol and redissolved in H_2_O.

### Preparation of the reduced AP site substrate

The protocol was adapted from previous studies [20]. N3–1 U iso G (450 pmol) was digested using 10 units of UDG in 20 μl reaction volume at 37°C for 1 h. UDG was removed through phenol-chloroform extraction, and the resulting N3–1 AP iso G was precipitated using isopropanol. Glycogen (Thermo Fisher Scientific, R0561) was added to a final concentration of 0.1 mg/ml to help form a compact DNA pellet. The purified N3–1 AP iso G was resuspended in 10 μl of H_2_O. A 2 M NaBH_4_ solution was prepared by adding 17 mg NaBH_4_ powder to ice-cold 225 μl 100 mM Tris–HCl (pH 8.0). This solution was made fresh shortly before every experiment and kept on ice until use. An aliquot of 41 μl 2 M NaBH_4_ solution was mixed with 220 μl ice-cold H_2_O (NaBH_4_ final concentration was 0.3 M) and immediately added to 10 μl N3–1 AP iso G. The mixture was kept on ice for 30 min, and 15 μl of 1 M HCl was added to quench the reaction. The pH of the mixture was determined using a pH strip and adjusted to 7 if necessary. Then, the N3–1 rAP (reduced AP) iso G was precipitated again and resuspended in 20 μl H_2_O. The final product could be added directly to 100 pmol nicked N3–1 for oligo swapping.

### Enzymatic method for isotopic labeling

To synthesize an isotopically labeled oligonucleotide, 1200 pmol of Ver 6 3′ U (Integrated DNA Technologies) and 1320 pmol of Ver 6 mass AG syn comp R (Integrated DNA Technologies) were first annealed in H_2_O. Then, five units of 3′-5′ exonuclease-deficient Klenow fragment DNA polymerase (NEB, M0212S), 15 nmol of each dNTP (NEB, N0446S) [or the required isotopically labeled dATP (Cambridge Isotope Laboratories, CNLM-6219-CA-20) and dGTP (Cambridge Isotope Laboratories, CNLM-6221-CA-20)], and 20 μl of 10× rCutSmart (NEB, B6004S) were added into the reaction to form a 200 μl reaction mix. The reaction was incubated at 37°C for 1 h and quenched by incubating at 80°C for 20 min. Then 300 units of BsoBI (NEB, R0586S) were added into the reaction mix to trim the 3′ end of the oligo at 50°C for 3 h. Then, 26 μl of 3 M KOH was added to the reaction mix, and the phosphodiester bonds between ribonucleotides and deoxyribonucleotides were broken by incubating at 50°C for 1 h. To neutralize the solution, 26 μl of 3 M HCl was mixed into the reaction mix. The pH of the mixture was determined using a pH strip and adjusted to 7 if necessary. Then, the isotopically labeled oligo was purified by phenol/chloroform purification and isopropanol precipitation with 0.2 μg/μl glycogen (Thermo Fisher Scientific, R0561).

To synthesize V7 isotopically labeled oligo, V7 3′ G (Integrated DNA Technologies) and Ver 7 mass AG syn comp R (Integrated DNA Technologies) were annealed. To synthesize a 5′A-labeled oligo, mass-labeled dATP (Cambridge Isotope Laboratories, CNLM-6219-CA-20), dUTP (NEB, N0459S), dCTP (NEB, N0446S), and dGTP (NEB, N0446S) were used. To synthesize a 3′-G-labeled oligo, dATP (NEB, N0446S), dUTP (NEB, N0459S), dCTP (NEB, N0446S), and mass-labeled dGTP (Cambridge Isotope Laboratories, CNLM-6221-CA-20) were used. The subsequent purification process was the same as in V6 oligosynthesis protocol.

### Preparation of *Xenopus laevis* HSS extracted from eggs

*Xenopus laevis* egg laying was induced by a priming injection of 100 U of pregnant mare serum gonadotropin at least 48 h before use and a boosting injection of 500 U of human chorionic gonadotropin (hCG) 16 h before use. Following the hormone injection, adult female *X. laevis* were placed in a 2 L water bath of 100 mM NaCl, 2 mM KCl, 1 mM MgCl_2_, 2 mM CaCl_2_, 0.1 mM ethylenediaminetetraacetic acid (EDTA), and 5 mM HEPES, pH 7.8, overnight at 17°C. The following morning, fresh eggs were squeezed from ovulating *Xenopus*.

*Xenopus* egg extract was prepared by centrifugal crushing of eggs and removal of the cytoplasmic layer. Freshly laid eggs were collected, dejellied with a 2% cysteine solution, and fractionated by centrifugation. The cytoplasmic layer was isolated and supplemented with 10 μg/ml each of leupeptin, pepstatin, and chymostatin; 20 μM cytochalasin-D; and an ATP regeneration mix (15 mM creatine phosphate, 2 mM ATP, pH 7.7, 2 mM MgCl_2_). CaCl_2_ was then added to the supplemented cytoplasm extract to a final concentration of 0.5 mM and incubated for 30 min to allow the extract to cycle to interphase. Then cycloheximide was added at a final concentration of 250 μg/ml to arrest the cytoplasm in interphase before high-speed centrifugation at 100 000 × *g* for 90 min to further fractionate the cytoplasm. The HSS was removed immediately after the centrifugation. Small aliquots of HSS were flash frozen in liquid nitrogen and stored at −80°C until use, and each aliquot was used only once.

### *In vitro* BER assay

For polyacrylamide gel experiments, reaction mixtures containing 0.75 μg/μl *Xenopus* HSS, 150 nM double-stranded DNA substrate, and 1× reaction buffer, which was prepared by mixing 1 part of extraction buffer [90 mM KCl, 30 mM Tris–HCl, pH 7.5, 2 mM ethyleneglycol-bis(β-aminoethyl)-N,N,Nʹ,Nʹ-tetraacetic acid, and 1 mM dithiothreitol] with 2 parts of supplementary buffer (30 mM HEPES–KOH, pH 7.5, 30 mM KCl, 16 mM MgCl_2,_ 3 mM dithiothreitol, 5 mM ATP, and 0.1 mM of each dNTP), were incubated at 25°C for 1 h and quenched by incubation at 80°C for 20 min. When using the plasmid substrates, the Xenopus HSS concentration was increased to 1.5 μg/μl.

Typically, 1.5 pmol of double-stranded DNA substrate and 10 μg of *Xenopus* HSS were used for analysis by denaturing polyacrylamide gel electrophoresis, while 36 pmol of DNA plasmid substrate and 360 μg of *Xenopus* HSS were used for mass spectrometry experiments. The reactions were incubated at 37°C for 1 h and stopped by incubating at 80°C for 20 min.

To assess the repair of the double-stranded oligonucleotide substrate, 1 pmol of the repaired product was digested with 2.5 units each of UDG (NEB, M0280S) and Ape1 (NEB, M0282S) (U substrate), or with four units of OGG1 (NEB, M0464S) (8-oxoG), or with 2.5 units of Ape1 (NEB, M0282S) (AP and rAP) in rCutSmart (NEB, B6004S) buffer. The resulting digested product was resolved by denaturing polyacrylamide gel electrophoresis. To assess the repair of the uracil plasmid substrate, 0.5 pmol of the repaired substrate was incubated with 2.5 units of UDG (NEB, M0280S) at 37°C for 30 min. Then, 5 units of NdeI (NEB, R0111S) were added to the reaction and incubated at 37°C for 1 h. The digestion product was resolved in an ethidium bromide-containing agarose gel. To assess the repair of rAP plasmid substrate, 0.5 pmol of the repaired substrate was directly incubated with 2.5 units of NdeI (NEB, R0111S) and electrophoresed as previously.

### Assay for BER in intact *X. laevis* eggs

Fresh eggs were squeezed out from ovulating *Xenopus*, washed with H_2_O, and dejellied. Subsequently, the eggs were washed five times with 1/3 MR (solution of Marc’s Modified Ringers, 100 mM NaCl, 2 mM KCl, 1 mM MgCl_2_, 2 mM CaCl_2_, 0.1 mM EDTA, 5 mM HEPES, pH 7.8, diluted 1:3 in H_2_O) and then transferred into an injection dish containing 2.5% Ficoll in 1/3 MR. Each egg received an injection of 5 nl of the BER substrate (5 μg/μl). The injected eggs were flash-frozen after 5, 10, 15, 30, 60, and 120 min.

To lyse the eggs, four volumes of lysis buffer mix (20 mM Tris–HCl, pH 7.6, 100 mM NaCl, 20 mM EDTA) were added to the frozen eggs. After vortexing for 1 min, the lysate was centrifuged at 17 900 × *g* at 4°C for 5 min. Subsequently, the supernatant was phenol-chloroform extracted three times. Every time after vortexing with phenol-chloroform, the sample was chilled on ice for 5 min before centrifugation. To precipitate the plasmid, 0.1 volume of 3 M sodium acetate and 0.7 total volume of isopropanol were added to the supernatant, which was then stored at −20°C for 1 h. After centrifugation at 17 900 × *g* at 4°C for 30 min, the pellet was dissolved in 1 ml TE buffer on a 65°C heat block with constant pipetting. Subsequently, 9 ml of buffer QBT (Qiagen, 12143) was added to the solution with constant shaking. The plasmid was then purified using a Midi-prep (Qiagen, 12143) column according to the manufacturer’s protocol.

### Determination of BER repair patch size by mass spectrometry

When magnetic streptavidin beads were not used, the procedure involved digesting 36 pmol of the BER product with 50 units of HaeII (NEB, R0107S) in a total reaction volume of 35 μl, buffered with BSA-free CutSmart buffer (50 mM potassium acetate, 20 mM Tris-acetate, 10 mM magnesium acetate, pH = 7.9). After incubation at 37°C for 2 h, 50 units of NdeI (NEB, R0111S) were added to the reaction mixture, which was incubated at 37°C for 9 h.

However, when magnetic streptavidin beads (Thermo Fisher Scientific, 65001) were employed, 36 pmol of the BER product was digested with 50 units of HaeII (NEB, R0107S) and PstI (NEB, R0140S) at 37°C for 2 h. Subsequently, 200 μg of streptavidin magnetic beads were washed three times in 100 μl of wash buffer (10 mM Tris–HCl, pH 7.5, 1 mM EDTA, and 2 M NaCl), and then resuspended in 100 μl of two-fold concentrated wash buffer. A mixture of 50 μl of the digestion product and 50 μl of H_2_O was added to the bead suspension. This mixture was placed on a rotor and incubated at 37°C for 1 h. The beads were then washed three times with wash buffer and three times with H_2_O. The beads were subsequently suspended in BSA-free CutSmart buffer, along with 60 units of NdeI (NEB, R0111S), in a reaction volume of 50 μl. The tube was placed on a rotor and incubated at 37°C for 9 h. The supernatant was collected for the following process.

To desalt the samples for mass spectrometry, 1 M triethylammonium acetate (TEAA) was added to the final product, achieving a final concentration of 0.1 M. The 10 μl C18 Ziptip (Sigma–Aldrich, ZTC18S096) was primed by rinsing three times with 10 μl 50% acetonitrile and three times with 10 μl 0.1 M TEAA. The sample was pipetted and flowed through the column 10 times. After that the column was rinsed three times with 0.1 M TEAA and deionized H_2_O. The sample was then eluted in 10 μl elution buffer (50% acetonitrile, 25 mM hexafluoroisopropanol, and 10 mM triethylamine). The elution buffer must be prepared just before use and kept on ice. Subsequently, the same sample was desalted using two more Ziptips, following the same procedure but using the previous eluate for the elution. The eluted material was analyzed in negative ion mode by electrospray ionization quadrupole time-of-flight mass spectrometry (Sciex 5600 + Qq time-of-flight) coupled with an Eksigent nanoLC 400 HPLC. The mobile phase A was 10 mM TEAA, and the mobile phase B was acetonitrile. The sample was injected (2 μl loop volume) at 8 μl/min in 50% phase A plus 50% phase B and ionized using a DuoSpray Ion source (ISVF-4500V, at 300°C, GS1 15, GS2 7, CUR 25, DP-90, and CE-10). The time-of-flight mass range was 900–1800 m/z, and the data were collected using Analyst TF v1.7.1. The measured molecular weight is calculated as (m/z + 1.008)*charge number.

To quantify the BER patch size distribution, the mass spectrum from when the sample entered the quadrupole to when the sample signal went away was averaged. Screenshots were made, and the target signal heights were measured and summed using ImageJ software. The relative proportion of each patch-size product was calculated by dividing the peak height of that product by the total peak height of all repair products.

### Preparation of cell-free extract

HEK 293 FT frozen cell stocks were thawed in a 37°C water bath and then added to 10 cm cell culture dishes with 10 ml of pre-warmed Dulbecco’s modified Eagle medium (DMEM) (Gibco, 11965-092) supplemented with 10% fetal bovine serum (Corning, 35-015-CV) and Gibco antibiotic-antimycotic (Gibco, 15240062). The cells were incubated overnight at 37°C in a 5% CO_2_ incubator to allow their attachment to the plates. The culture medium was changed the following day and every 2 days thereafter. When the cells reached 80% confluency, trypsin-EDTA treatment (Sigma–Aldrich, T4049-100ML) was utilized to detach the cells, and 4.4 × 10^6^ cells were seeded in another 10 cm cell culture dish.

Whole-cell extracts (WCE) were prepared after the cells had been passaged eight times and reached 80% confluency. The cells were trypsinized, transferred to a 15 ml centrifuge tube, and centrifuged at 600 × *g*, 4°C for 5 min in a free-angle centrifuge. The cell pellet was washed once with phosphate-buffered saline (PBS) and then centrifuged again. Subsequently, the cells were resuspended in buffer A (200 mM KCl and 10 mM Tris–HCl, pH 7.8; 80 μl per 10 cm cell culture dish). The suspension was combined with an equal volume of buffer B (600 mM KCl, 2 mM EDTA), and the tube was placed on a rotor in a 4°C cold room for 1.5 h. The lysate was centrifuged at 16 000 × *g* for 10 min at 4°C. The resulting supernatant was collected and divided into 20 μl aliquots. These aliquots were flash-frozen in liquid nitrogen and stored at −80°C. The protein concentration of the WCE was determined using the Bradford reagent (Bio-Rad, 5000006) according to the manufacturer’s protocol.

### *In vitro* BER assay

Assays using WCE were carried out in reactions containing 45 mM 4-(2-Hydroxyethyl)-1-piperazineethanesulfonic acid-KOH, pH 7.5, 60 mM KCl, 5 mM MgCl_2_, 0.4 mM EDTA, 1 mM dithiothreitol, 2 mM ATP, 1 mM nicotinamide adenine dinucleotide, 100 μg/ml bovine serum albumin, 20 μM each of dATP, dTTP, dCTP, and dGTP, 1.5 μg/μl of extract, and 150 nM of the DNA substrate. The purification of the BER product and MS procedures were the same as for the *Xenopus* experiments.

### Cell cycle synchronization

HEK293 FT cells were synchronized at the G_1_/S boundary using a double thymidine block. Briefly, 1 × 10^6^ cells were seeded into 10-cm tissue culture dishes containing DMEM (Gibco, 11965-092) supplemented with 10% fetal bovine serum and antibiotics. After 24 h, thymidine (Sigma, T1895), prepared as a 100 mM stock in DMSO, was added to the medium to a final concentration of 2.5 mM. Cells were incubated for 17 h at 37°C in a humidified 5% CO_2_ incubator. The thymidine-containing medium was then removed, and cells were washed three times with PBS to remove residual thymidine. Fresh complete DMEM was added, and cells were released for 9 h. A second 2.5 mM thymidine block was then applied for an additional 17 h to arrest cells at the G_1_/S transition. WCE were subsequently prepared as described previously.

To validate synchronization and determine cell cycle distribution, 1 × 10^6^ cells were harvested and pelleted by centrifugation at 600 × *g* for 5 min. The pellet was resuspended in 1 ml of room temperature PBS and slowly added to 4 ml of −20°C absolute ethanol while vortexing. Cells were fixed at −20°C for 15 min, then pelleted again and rehydrated in 5 ml of room temperature PBS. After another round of centrifugation, the cells were resuspended in 500 μl of propidium iodide staining solution (IHCWorld, IW-1403) containing 100 μg/ml RNase A (Sigma–Aldrich, R4642). The staining was carried out for at least 20 min at room temperature in the dark. DNA content was analyzed using an LSR Fortessa flow cytometer (BD Biosciences), and data were used to assess cell cycle distribution.

### Immunodepletion

To achieve immunodepletion of hFen1 in 1 mg WCE (60 μl under our experimental conditions), 50 μl of Protein A Dynabeads (Invitrogen, 10001D) and 25 μl of hFen1 antibody (Proteintech, 14768-1-AP) were utilized. The beads were washed three times, each time with 100 μl of PBS-T (PBS with 0.02% Tween 20), and subsequently resuspended in 25 μl of PBS-T. Anti-hFen1 antibody (25 μl) was added to the bead suspension and incubated at room temperature for 30 min with rotation. The beads were then washed three times using PBS-T and resuspended in 60 μl WCE. The mixture was incubated at 4°C for 1 h with rotation. Finally, the WCE was collected, flash-frozen in liquid nitrogen, and stored at −80°C.

### Western blotting

An equal volume of 2× Laemmli buffer (4% SDS, 10% 2-mercaptoethanol, 20% glycerol, 0.02% bromophenol blue, and 0.125 M Tris–HCl, pH 6.7) was added to WCE and boiled for 5 min. Protein samples were resolved by electrophoresis in 10% SDS–polyacrylamide gels and transferred to hydrophilic polyvinylidene fluoride membranes (Sigma, IPFL20200). The membranes were blocked in 5% non-fat milk in TBS-T (20 mM Tris, 150 mM NaCl, and 0.1% Tween 20, with a pH of 7.4) for 1 h at room temperature. Rabbit polyclonal anti-hFen1 antibody (1:500, Proteintech, 14768-1-AP), rabbit polyclonal anti-xFen1 antibody (1:1000, see the “Acknowledgments” section), rabbit polyclonal anti-hPolβ antibody (1:250, Novus, NBP2-38600), rabbit polyclonal anti-GAPDH antibody (1:1000, Rockland, 600-401-A33), and IRDye 800CW labeled goat anti-rabbit IgG (1:10 000, LI-COR, 926-32211) were diluted in 1% non-fat milk in TBS-T. The blocked membrane was incubated with indicated primary antibody at 4°C overnight. The blots were then washed four times with TBS-T, 5 min each time, and incubated with IRDye 800CW labeled goat anti-rabbit IgG (1:10 000, LI-COR, 926-32 211) at room temperature for 30 min. The membrane was washed four times with TBS-T, 5 min each time, and imaged on a LI-COR Odyssey.

### Data Analysis

All time-scale repair efficiency experiments were analyzed using two-way ANOVA implemented in GraphPad Prism. Time was specified as the matching factor to account for continuous sampling across time points. Multiple comparisons of repair efficiencies between different substrates at the same time point were corrected using Tukey’s post hoc test.

Product proportion data were analyzed within a compositional data framework. To account for the simplex constraint (all components are non-negative and sum to one), we applied Dirichlet regression using the R package DirichletReg. A small pseudocount was added to zero values to place all observations strictly inside the open simplex, followed by normalization of each sample to unit sum. The model included the experimental grouping factor as a predictor, and likelihood-ratio tests were used to assess global differences in product composition between groups. Predicted group-wise compositions were obtained from the fitted model and visualized as stacked bar plots. For component-wise inference, each product was further evaluated using beta regression (R package betareg), and multiple testing across products was controlled using the Benjamini–Hochberg false discovery rate (FDR) procedure.

All datasets analyzed by Dirichlet regression showed significant global differences in product composition between experimental groups.

## Results

### Base excision repair in *Xenopus laevis* egg extracts

**Figure 1.**
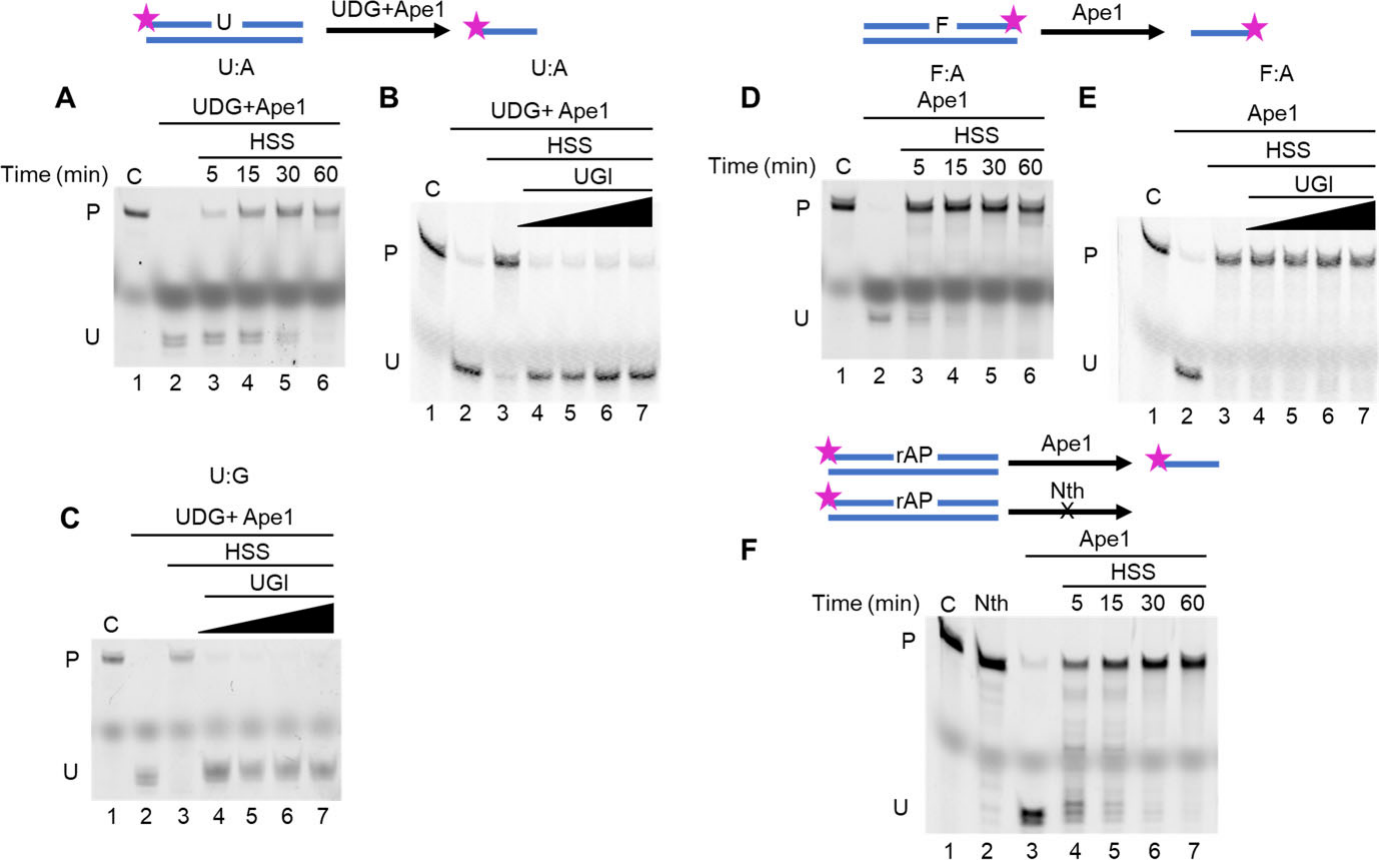
*Xenopus* HSS has robust BER activity. (**A**) The U:A substrate was incubated with *Xenopus* HSS for the indicated times, then digested with UDG + Ape1 to nick the unrepaired portion. Full-length substrate (indicated by C above lane 1), UDG + Ape1 digested substrate (lane 2), and UDG + Ape1 digested, HSS-repaired samples (lanes 3–6) were electrophoresed in a denaturing polyacrylamide gel, then imaged using a fluorescent imager. HSS was pre-incubated with uracil glycosylase inhibitor (UGI) to inhibit endogenous UDG activity before adding the U:A substrate (**B**) or the U:G substrate. (**C**) The F:A substrate was incubated with *Xenopus* HSS for the indicated times, without (**D**) or with (**E**) added UGI (0.5, 1, 2, 3 units/μl). Ape1 was added where indicated to cleave unrepaired molecules. (**F**) The LP-BER substrate rAP was incubated with *Xenopus* HSS. Nth was used as a control for AP site reduction since Nth cannot digest rAP, while Ape1 can. For all panels, C indicates the starting substrate, P the repaired product, and U the unrepaired substrate or a repair intermediate. F, tetrahydrofuran; rAP, reduced AP site.

We sought initial insights into the BER machinery in *Xenopus* by quantifying the repair of several key lesions using linear duplex DNA substrates. For this purpose, uracil served as a BER substrate that has been well characterized in many other systems, and duplex substrates with centrally located U:A or U:G pairings were employed. The former represents a product of DNA replication in which dUMP was incorporated instead of dTMP [4]; the latter represents the product of cytosine deamination in the DNA template, which threatens mutation if left unrepaired [21]. These were installed in 52-mers with a 5′ fluorescent tetramethylrhodamine (TAMRA) label in the uracil-containing strand. Complete repair was confirmed by the resistance of the products to cleavage by UDG and the Ape1 AP endonuclease (Fig. 1A). *Xenopus* HSS extract had robust repair activity for the U:A substrate, and in pilot experiments significant dilution (40- to 80-fold) of the extract was required to bring the reaction into the linear range for time dependence. Under these conditions, the repair was essentially complete within 60 min (Fig. 1A). That the repair was carried out by BER was confirmed by its complete inhibition by the addition of Ugi (Fig. 1B), a potent and specific protein inhibitor of UDG [22].

Similar results were obtained for the U:G substrate: complete repair in <60 min and total inhibition in the presence of Ugi (Fig. 1C). These results also indicate that Ugi-resistant uracil glycosylases such as SMUG [23] are not responsible for the repair of uracil by the HSS, regardless of the opposite base (also see kinetic analysis below).

We tested a substrate known to require LP-BER, a synthetic abasic site (tetrahydrofuran, F) [24] paired with A in the same duplex DNA sequence. Unlike an ordinary AP site, the F residue is resistant to the 5′-dRp lyase of Polβ [25]. In this case, the resistance of the product to cleavage by Ape1 alone confirmed the repair. Repair of the F:A substrate was at least as rapid as that of U:A, being completed in <30 min under our conditions (Fig. 1D). In contrast to the U:A substrate, this repair reaction was totally resistant to Ugi (Fig. 1E), as expected because no glycosylase step was required.

AP residues are in rapid equilibrium between the furanose (ring) form and the aldehyde (chain) form, with the latter being the substrate for AP lyase activities [26–28]. The F substrate mimics the former [24], and a stable mimic of the latter can be generated by chemical reduction [20], changing the aldehyde to an alcohol and preventing ring closure [29]. We generated a reduced AP site (rAP) substrate by using NaBH_4_ reduction after removal of uracil from the U:A substrate by UDG. This material remains sensitive to cleavage by Ape1, but it becomes resistant to AP lyases [30] such as Nth protein (Fig. 1F). The rAP:A substrate was also rapidly repaired by HSS in ≤30 min (Fig. 1F). These results set the stage for more detailed experiments to follow.

**Figure 2.**
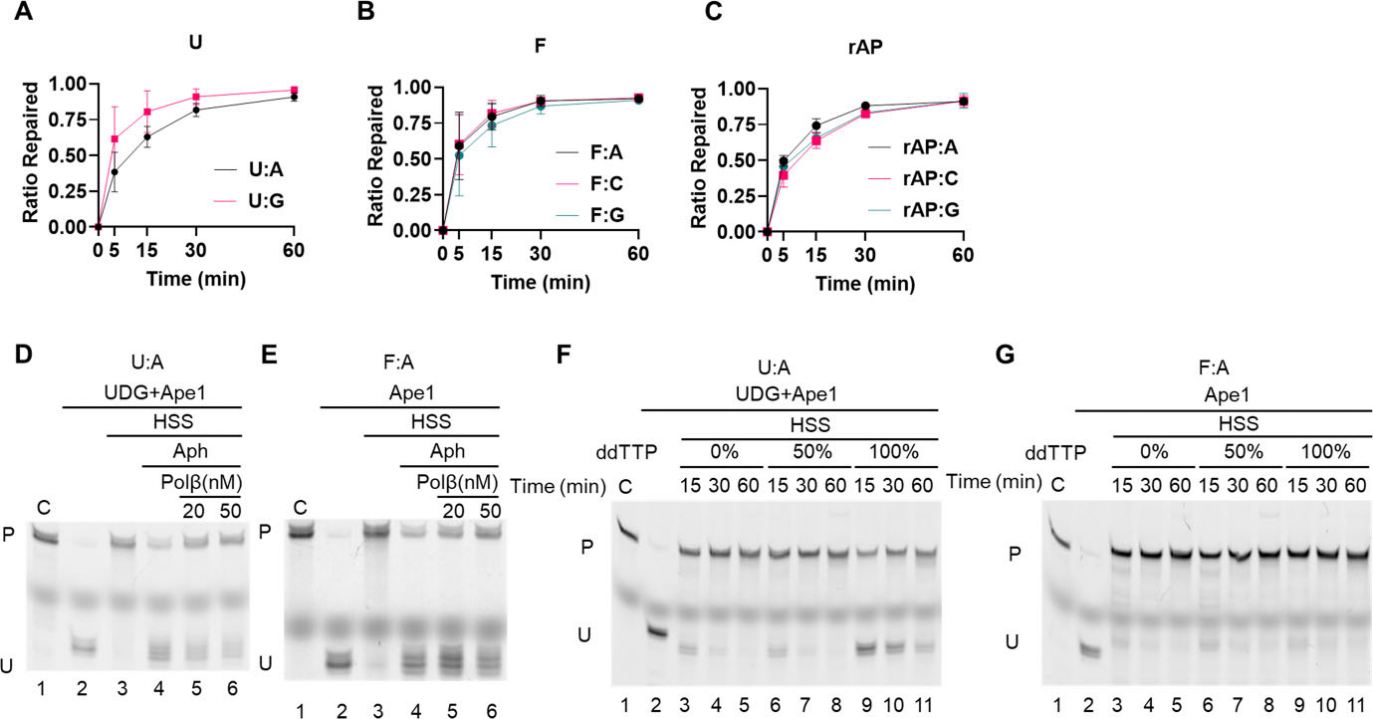
Repair rates, base pairing, and contributions of DNA repair polymerases in the HSS. (**A–C**) Linear duplex oligonucleotide substrates were incubated with *Xenopus* HSS for the indicated times, and the repaired products were resolved by denaturing polyacrylamide gel electrophoresis as in Fig. 1, with the fraction repaired was quantified by ImageJ. Each data point resulted from three biological replicates, and the data shown are the mean ± standard deviation (SD). (A), U paired with A or G; (B), F paired with A, C, or G; (C), reduced AP site (rAP) paired with A, C, or G. *n* = 3 biological replicates, and the data are means ± SD. (**D, E**) Aphidicolin sensitivity of BER in *Xenopus* HSS. Where indicated, the HSS was pre-incubated with aphidicolin (100 μg/ml) to inhibit replicative DNA polymerases, with or without added Polβ, incubated with the substrate, and then analyzed for repair as in Fig. 1. (D), U:A substrate, with UDG + Ape1 to cleave unrepaired molecules. (E), F:A substrate, with Ape1 to cleave unrepaired molecules. (**F, G**) Effect of added ddTTP on the repair reactions. (F) Repair of the U:A substrate; (G), repair of the F:A substrate. C, control; P, repaired product; U, unrepaired substrate or repair intermediate.

The opposite base in each substrate was varied, using molecules with U:A and U:G; F:A, F:C, and F:G; and rAP:A, rAP:C, and rAP:G sites. The abasic pairings with C reflect lesions due to G depurination or repair intermediates during BER of 8-oxoG, for example [31]. For the U substrates (Fig. 2A), the F substrates (Fig. 2B), and the rAP substrates (Fig. 2C), the rates were essentially unaffected by the opposite base.

### BER contributions of DNA polymerases in *X. laevis* egg extracts

The contribution of different DNA polymerases to BER in the HSS system was assessed initially by employing inhibitors. A proteomic analysis indicated that the HSS contains high levels of the replication polymerases, DNA polymerase α (Polα), Polδ, and Polε, and very little Polβ [32]. The quantitative dominance of the replication polymerases likely reflects that *Xenopus* eggs are poised to initiate rapid DNA replication upon fertilization [33], but it also prompts questions about how BER is conducted in *X. laevis* eggs. Consistent with this profile, the addition of aphidicolin, which inhibits Polα, Polδ, and Polε, but not Polβ, eliminated most detectable repair of both U:A (Fig. 2D) and F:A (Fig. 2E). However, for the U:A substrate in the presence of aphidicolin, supplementing the HSS with recombinant human Polβ (to a final concentration comparable to Polδ) restored complete BER of U:A (Fig. 2D). This contrasted with results for the F:A LP-BER substrate, for which Polβ addition in the presence of aphidicolin only partially restored complete BER (Fig. 2E). The use of the human Polβ was deemed appropriate because of its high degree of conservation compared to the *Xenopus* ortholog, which was not available. Furthermore, a structure prediction for *X. laevis* Polβ using AlphaFold showed a remarkably good fit to the co-crystal structure of the human enzyme bound to a DNA substrate (Supplementary Fig. S1).

The options were more limited in probing the contribution of the Polβ presented in the HSS. Dideoxynucleotides are the best-characterized inhibitors for Polβ, acting competitively with normal dNTPs, while the replication DNA polymerases are relatively resistant [34]. We chose 2′, 3′-dideoxy-TTP (ddTTP) for probing the reactions with the U:A and F:A substrates, as dTTP would be the nucleotide required for both SP-BER and LP-BER. Increasing the proportion of added ddTTP from 0% to 100% showed at most a delaying effect for the U:A substrate, with most repair completed in 60 min (Fig. 2F). There was very little effect of ddTTP on F:A repair (Fig. 2G). Again, this result was consistent with the paucity of Polβ in the HSS. The differences between the results for U:A and F:A indicate a difference in polymerase usage, with a lesser role for Polβ in the latter. The incomplete effects of added ddTTP likely also reflect the synthesis of dNTPs by the HSS supported by ATP production in the system.

### DNA repair synthesis tracts generated by *Xenopus* egg extracts

**Figure 3.**
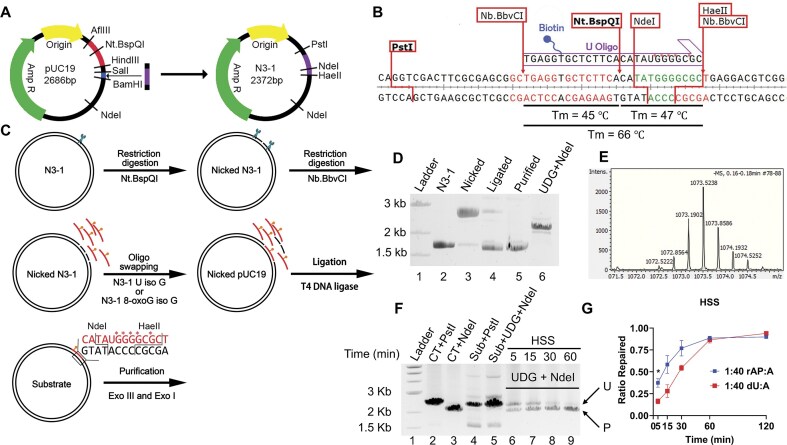
The isotopic labeling plasmid approach to determine BER patch size. (**A**) The target DNA fragment for “oligo swapping” was inserted into the multicloning site of pUC19, and one of the Nt.BspQI sites was deleted. (**B**) The DNA sequence flanking the target site, indicating the cut sites of key restriction enzymes. The incoming biotin-labeled synthetic oligonucleotide is shown above the target sequence; the *G indicates isotopically labeled dGMP residues (fully substituted with ^13^C and ^15^N). The segment highlighted in green is used for mass spectrometry analysis. (**C**) Overview of the oligo swapping procedure (see the “Materials and methods” section for details). (**D**) Stepwise products from the substrate synthesis procedure in panel (C) were loaded on an ethidium bromide-containing agarose gel (lanes 2–5) and subjected to electrophoresis. A DNA ladder was loaded in lane 1. To determine the oligo swapping efficiency, the plasmid substrate was digested with UDG and NdeI (lane 6): removal of U prevents cleavage by NdeI. (**E**) Mass spectrometry analysis confirming the target fragment released from plasmid substrate by NdeI and HaeII. (**F**) A sample ethidium bromide-containing agarose gel image showing the repair product of plasmid substrate by *Xenopus* HSS at the indicated time points, with the repaired substrate susceptible to UDG and NdeI cleavage (lanes 6–9). A DNA ladder was loaded in lane 1. Native pUC19 was digested with PstI (lane 2) or NdeI (lane 3) for size markers. Plasmid substrate was digested with PstI (lane 4) or UDG + NdeI (lane 5) to confirm the presence of uracil. P, repaired product; U, unrepaired substrate. (**G**) Repair rates for U:A and rAP:A substrates incubated with *Xenopus* HSS. Gel images were quantified using ImageJ. * *P* < .05. *n* = 3 biological replicates and the data are means ± SD.

The contributions of different DNA polymerases to BER in the HSS were further explored by measuring the length of the repair synthesis tracts. The methodology for this approach is summarized in Fig. 3. Briefly, a modified (and shortened) pUC19 plasmid (Fig. 3A) was engineered to allow the installation of an oligonucleotide containing mass-labeled [^13^C, ^15^N]dGMP residues on the immediate 3′ side of a uracil lesion (Fig. 3B). The replacement of these residues by normal-mass dGMP during DNA repair allows determination of the repair “patch size” without the use of DNA mismatches or chemically modified residues, which might alter the repair reactions. An “oligonucleotide swapping” procedure (Fig. 3C) was developed to maximize the efficiency of this process, and the expected products were confirmed (Fig. 3D and E).

When treated with UDG, the substrate was not cleaved by NdeI (Supplementary Fig. S2), which provided an assay for the repair. The optimal amount of HSS for this assay was established in pilot experiments (example shown in Supplementary Fig. S3). Complete repair of the mass-labeled plasmid substrate by HSS was confirmed using UDG and NdeI. The result showed that BER was complete in 60 min (Fig. 3F). As was done with the linear substrate, a substrate expected to require LP-BER was produced by sequential treatment with UDG and chemical reduction, yielding an rAP site. Comparison of the repair kinetics for the plasmid U:A and rAP:A substrates showed significantly faster repair for the abasic substrate (Fig. 3G), perhaps due to the extra DNA glycosylase step needed for U:A.

**Figure 4.**
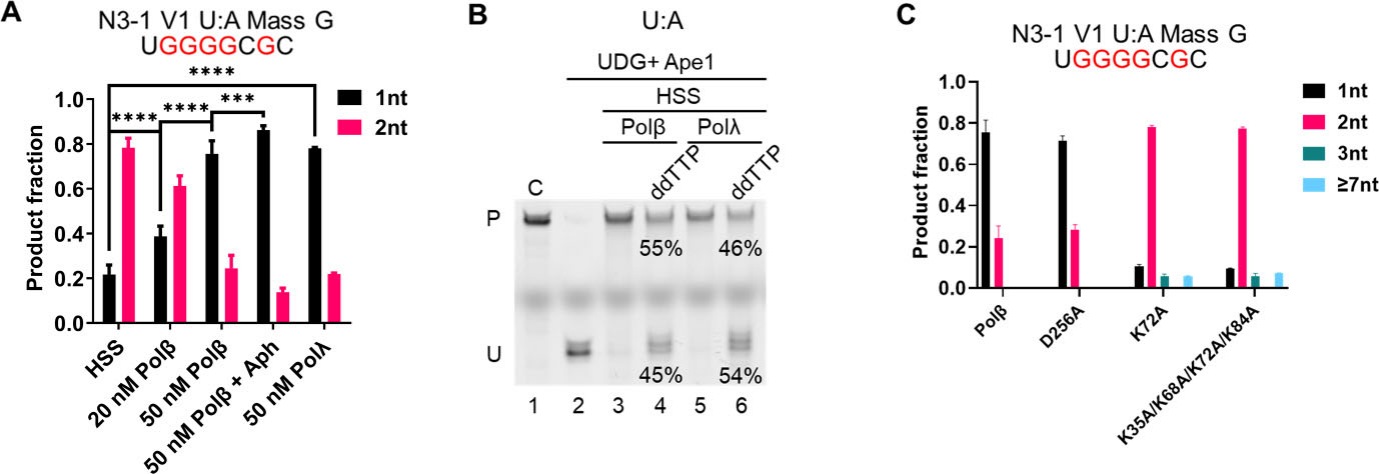
DNA polymerase dictates BER patch size. (**A**) The BER patch size for a U:A plasmid substrate generated by *Xenopus* HSS, or HSS supplemented with the indicated proteins and inhibitors. Aph, aphidicolin (100 μg/ml). *n* = 3 independent experiments and the data are means ± SD. *** indicates *P *< .001, **** indicates *P *< .0001. (**B**) Effect of ddTTP in the presence of added Polβ or Polλ. Incubation time was 60 min. Treatment with UDG and Ape1, followed by electrophoresis on a denaturing polyacrylamide gel, revealed fully repaired (P) and unrepaired (U) molecules. (**C**) Effect of Polβ polymerase and dRp lyase activities on BER patch size in the *Xenopus* HSS.

The isolation of oligonucleotides containing the repaired segments of the plasmid allowed the repair tracts to be determined by mass spectrometry. In strong contrast to what is typically observed in human (and mouse) cell extracts [35–38], the BER products generated by the *Xenopus* egg HSS showed that possible SP-BER accounted for only ∼20% of the total, with the remaining ∼80% all having 2-nt repair patches (Fig. 4A). Supplementing the extract with Polβ shifted the products to 1-nt as a function of the added enzyme, reaching ∼75% 1-nt patch size when the amount of added enzyme (50 nM) was estimated to be similar to the levels for Polα, Polδ, and Polε in the HSS (Fig. 4A). The addition of aphidicolin to the Polβ-supplemented reactions shifted the products slightly more of the 1-nt species (Fig. 4A). In the absence of aphidicolin, adding full-length Polλ instead of Polβ also shifted the products to 1-nt (Fig. 4A). The role of the added enzymes in repair was confirmed by replacing dTTP with ddTTP, resulting in only ~50% repair (Fig. 4B). The remaining repair in those cases likely reflects two things: the presence of dTTP in the HSS, which would compete with ddTTP for Polβ and Polλ, and the activities of Polα, Polδ, and Polε, which are much more resistant to ddTTP. In addition, we also tested the BER patch size on a U:G substrate. There was no difference between the U:A and the U:G substrates in the pattern of the resulting BER patch sizes (Supplementary Fig. S4).

We explored the contribution of individual Polβ functions in the supplemented HSS system, using the relaxed U:A plasmid substrate. Three engineered enzymes were assessed: the DNA synthesis-defective D256A protein; an enzyme defective in the main 5′-dRp lyase residue, K72A; and a derivative with three additional, minor lyase-active residues changed, the K35A/K68A/K72A/K84A protein [39]. The synthesis-defective protein generated a repair patch pattern almost indistinguishable from the wild-type enzyme (Fig. 4C). In contrast, both lyase-defective enzymes produced repair patches similar to those seen with non-supplemented HSS, although with small amounts of longer (3-nt or ≥7-nt) patches (Fig. 4C). These results indicate that only the lyase activity is required for the SP-BER we have detected.

### Influence of sequence context and DNA tertiary structure on repair patch size

The possible effect of tertiary DNA structure was addressed by using DNA gyrase to maximize the negative supercoils in the original plasmid substrate (see Fig. 3). Repair intermediates were generated by prior treatment with UDG alone or with both UDG and Ape1 protein.

**Figure 5.**
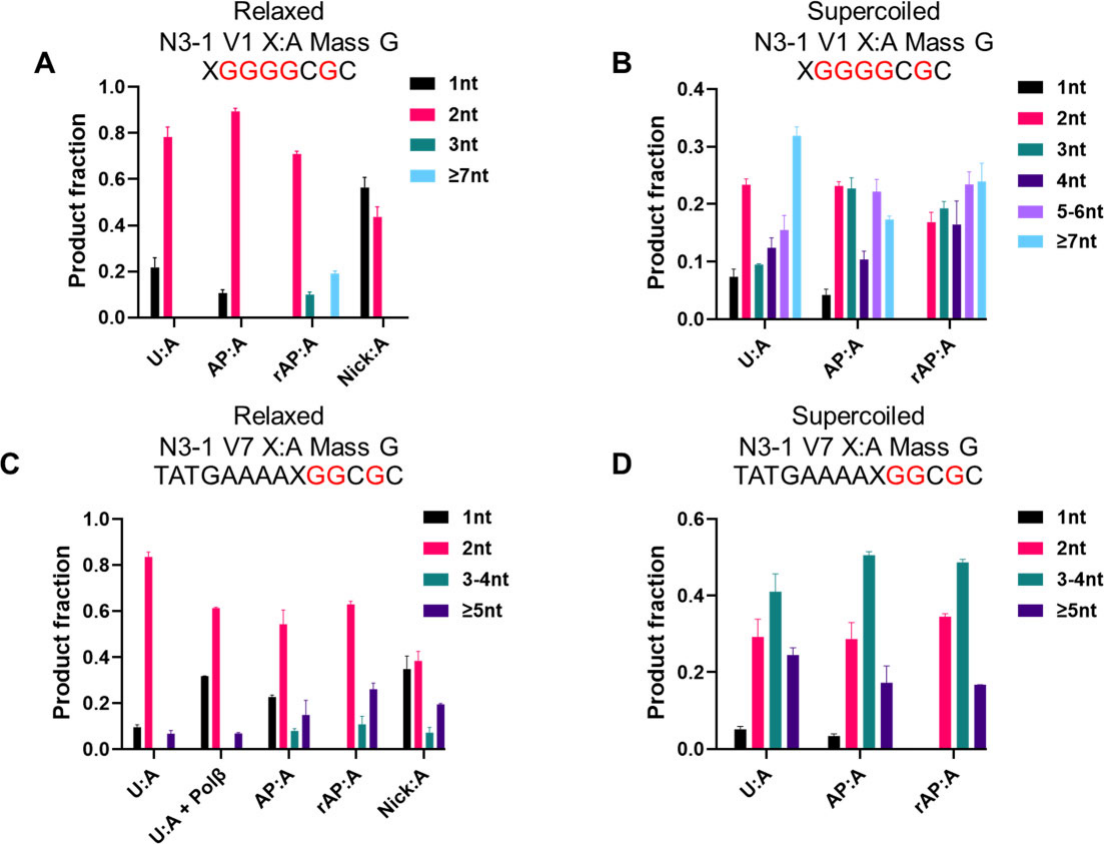
Effect of downstream sequence and topological state on BER patch size. Various lesions in (**A**) relaxed N3–1 V1 plasmid substrate, (**B**) supercoiled N3–1 V1 plasmid substrate, (**C**) relaxed N3–1 V7 plasmid substrate, and (**D**) supercoiled N3–1 V7 plasmid substrate were tested. Polβ final concentration was 50 nM. *n* = 3 independent experiments, and the data are means ± SD.

With a relaxed plasmid, even in the absence of added Polβ, pretreatment with both UDG and Ape1 increased the 1-nt product to almost 60% of the total, with the remainder accounted for by 2-nt repair patches (Fig. 5A). In contrast, pretreatment with UDG alone did not significantly change the repair patch size distribution (Fig. 5A). However, the highly supercoiled U plasmid displayed a significantly altered repair patch series, with <10% 1-nt product, only ∼25% 2-nt product, and the remaining two-thirds a series of longer patches out to ≥7-nt, which constituted ~33% of the total (Fig. 5B). In addition, supercoiling had a similar impact on the repair patch size distribution for the AP and rAP substrates (Fig. 5B and D).

The preceding patch-size data were all obtained for a single, G-rich sequence on the 3′ side of the DNA lesion. To test the possible sequence dependence of the extent of DNA repair synthesis, we used an alternative method to generate various mass-labeled oligonucleotides enzymatically (Supplementary Fig. S5). These oligonucleotides were installed in the corresponding plasmid molecules, which were incubated with HSS, followed by mass spectrometric analysis. The V7 substrate, with the 3′ runs of G interrupted, generated repair products that still showed only ∼10% SP-BER, with ∼80% 2-nt patches, with the small remaining amount showing patch lengths of ≥5-nt (Fig. 5C). Another version, with even less GC content 3′ of the lesion, gave only slightly more 1-nt product (∼25%), but a series of longer patches out to ≥8-nt, the latter accounting for >30% of the total (version V6 shown in Supplementary Fig. S6). Clearly, the sequences downstream of a repair site can strongly influence the amount of DNA repair synthesis, and these longer products underscore the likely contribution of the replication DNA polymerases to BER in this system.

We attempted to assess the contribution of xFen1 to BER in *Xenopus* HSS by immunodepletion. Anti-xFen1 polyclonal antiserum and recombinant xFen1 protein were kindly provided by the laboratory of Makoto Nakanishi [40]. Unfortunately, the antiserum proved ineffective in our immunodepletion protocol (Supplementary Fig. S7). We were also unable to use a commercial anti-hFen1 antibody to deplete xFen1 from the HSS, although that antibody was very effective in removing human Fen1 from a WCE of HEK293 cells (see below).

### 5′-excision during *Xenopus* BER

**Figure 6.**
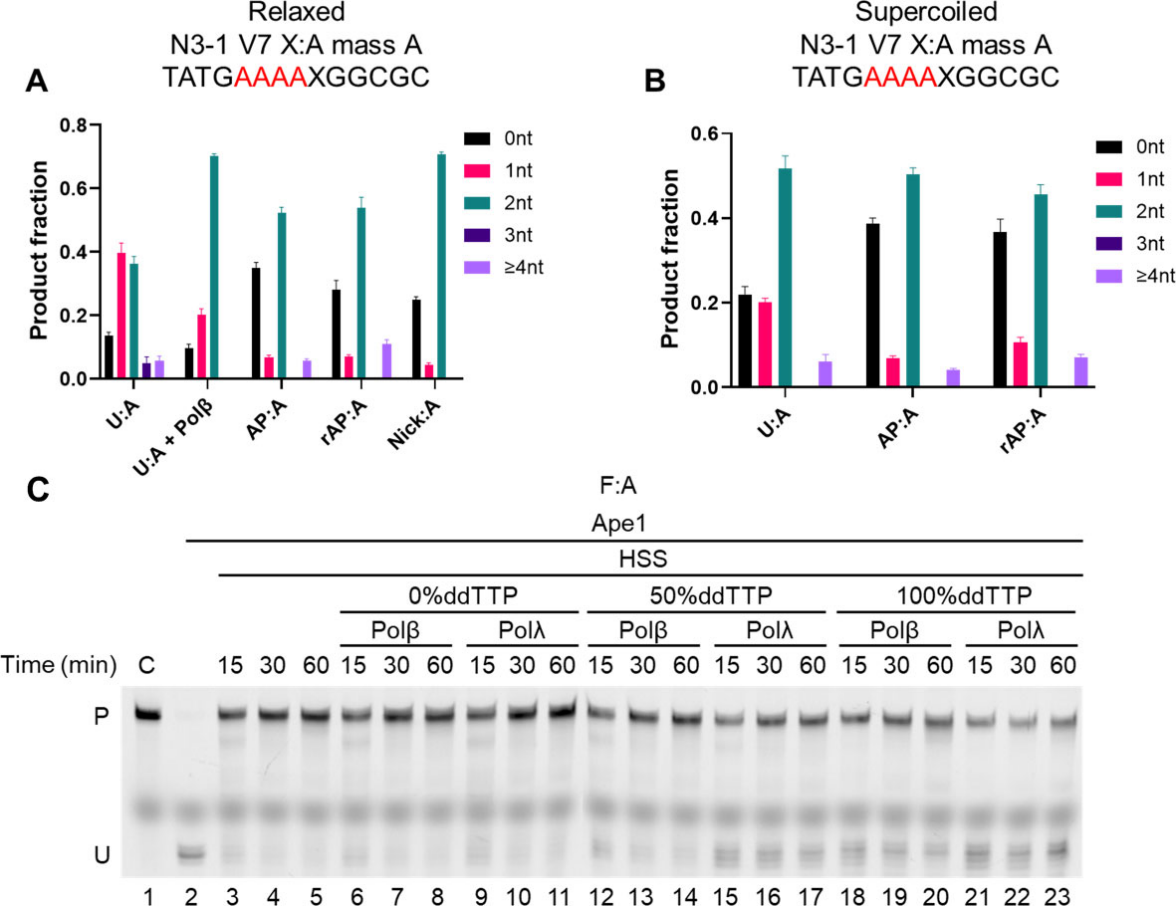
Upstream (5′) excision is a common event in *Xenopus* HSS BER. 5′ excision gap size of relaxed (**A**) and supercoiled (**B**) N3–1 V7 plasmid substrate with the indicated lesions. Polβ final concentration was 50 nM. (**C**) Dead-end products generated by Polβ and Polλ in the presence of ddTTP. *n* = 3 independent experiments and the data are means ± SD. The F:A oligonucleotide substrate was incubated with *Xenopus* HSS supplemented with 50 nM Polβ or Polλ in reaction buffers containing 0%, 50%, or 100% ddTTP (replacing dTTP) for the indicated times. The products were digested with Ape1 and loaded on a denaturing polyacrylamide gel (lanes 6–23). Full-length substrate (lane 1) and digested substrate (lane 2) were included as markers. Lanes 3–5, repair of F:A by *Xenopus* HSS without any supplementation.

We also used mass spectrometry to assess possible excision upstream (5′) of the lesion site during BER. Initially, this approach was planned as a control, but a recent report [11] suggested that 5′-excision may be a common event in BER mediated by mammalian cell extracts. In that case, there were base mismatches positioned near the lesion site, which was intended to allow excision tracts to be determined by conversion of the lesion-strand nucleotide to the template-strand nucleotide, analyzed after expansion of the recovered DNA in bacteria and DNA sequencing [11]. Since the presence of mismatches might alter the excision, or attract other DNA repair activities, our approach provides an important alternative. Although 5′ excision during BER has not been previously reported for *Xenopus*, we found that ∼80% of the HSS repair products for a U:A substrate were accompanied by 5′ excision tracts of 1–2 nt, and <10% with 5′ excision tracts of ≥3 nt (Fig. 6A). Only a minority (∼15%) of the repair products lacked any 5′ excision (Fig. 6A).

We examined the influence of added Polβ and different BER substrates on the 5′ excision pattern (Fig. 6A). Unexpectedly, the addition of 50 nM Polβ increased the proportion of 2-nt 5′ excision to ∼70%, with nothing longer detectable. Converting U to an AP site gave an almost bimodal pattern, with ∼35% having no 5′ excision and 50% with 2-nt excision. Interestingly, the LP-BER substrate rAP:A showed a similar bimodal pattern, while converting the AP site to the cleaved form shifted the 5′-excision products to ∼70% 2-nt (Fig. 6A).

The foregoing experiments were all performed on plasmids with limited DNA supercoiling (or in the case of the nicked substrate, none). Using recombinant DNA gyrase to negatively supercoil the AP:A and rAP:A substrates had relatively little effect on the 5′ excision patterns, perhaps increasing the fraction with no 5′ excision (Fig. 6B). For the U:A substrate, supercoiling increased the amount of 2-nt excision, mostly at the expense of the 1-nt product (Fig. 6B).

To examine the ability of the 5′ excision activity to remove aberrant 3′ termini, we supplemented the HSS with Polβ or Polλ and carried out repair reactions in the presence of increasing amounts of ddTTP. The incorporation of ddTMP by these enzymes generates 3′ termini that would not support further DNA repair synthesis unless they were first removed by excision toward the 5′ end. The results (Fig. 6C) show that, at the highest level of added ddTTP, a nicked intermediate accumulated by 15 min, the amount of which was not diminished by continued incubation for a further 45 min. As was seen in Fig. 4B, a significant amount of complete repair was observed in the presence of ddTTP, likely due to the presence or production of dTTP in the HSS. The lack of further processing of the nicked intermediate indicates that the 5′ excision activity does not remove the incorporated ddTMP, resulting in a dead-end product (Fig. 6C).

### Contrasts between the *X. laevis* and mammalian BER systems

**Figure 7.**
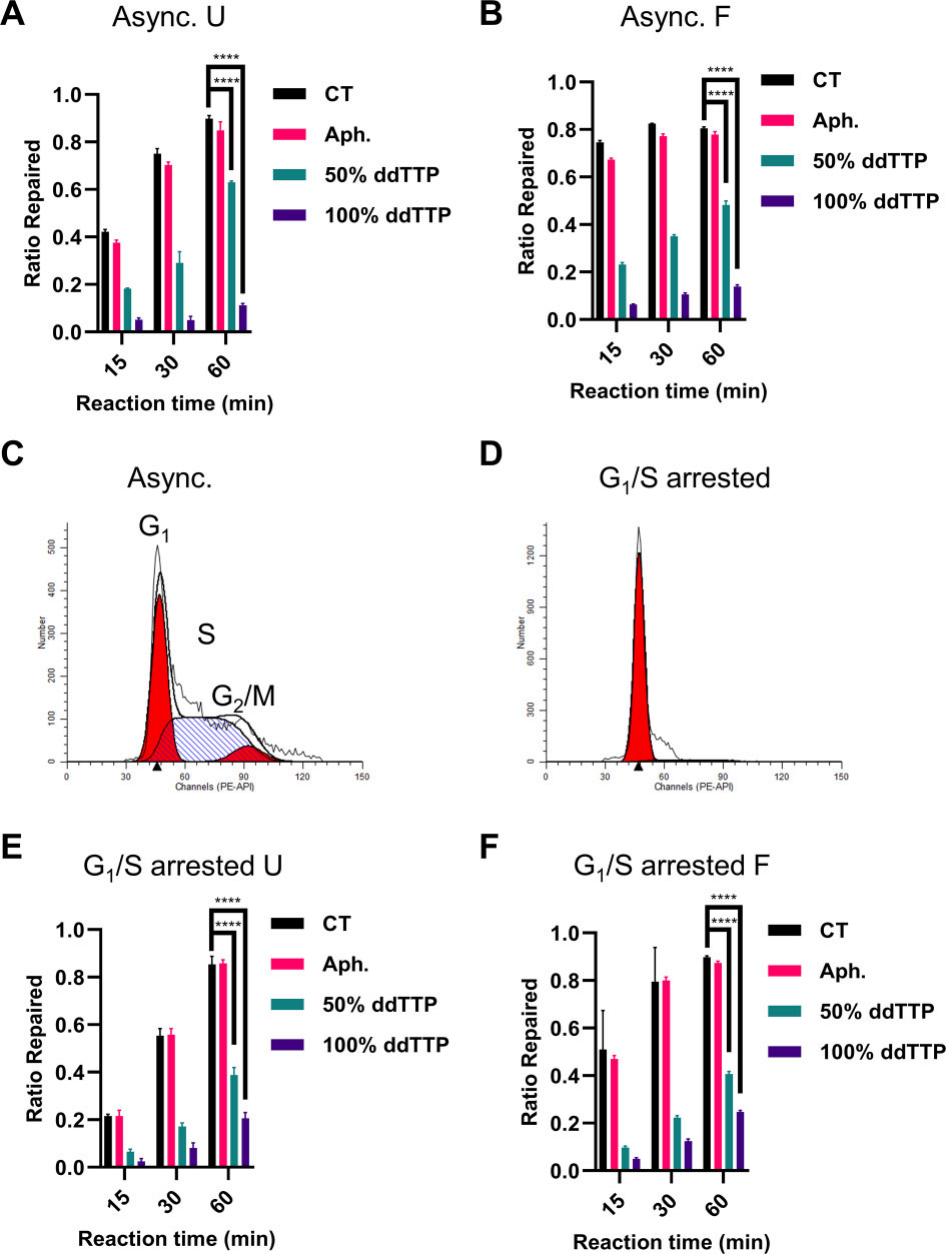
Polβ actively participated in the BER of G_1_/S synchronized and asynchronous HEK293 FT WCE. The U:A (**A**) or F:A (**B**) oligo substrate was incubated with asynchronous HEK293 FT WCE supplemented with aphidicolin (100 μg/ml) or reaction buffers containing 50% or 100% ddTTP for 1 h. The ratio of repaired products was quantified by ImageJ. Cell cycle profiles of asynchronous (**C**) and double thymidine synchronized **(D)** HEK293 FT cells. The sensitivity of WCE made from synchronized HEK 293 FT cells to aphidicolin (100 μg/ml) and ddTTP was determined using the U:A (**E**) or F:A (**F**) oligo substrate as in panels (A) and (B). *n* = 3 independent experiments and the data are means ± SD. *** indicates *P *< .001. **** indicates *P *< .0001. Aph, aphidicolin. Async, asynchronous.

In contrast to our results with the frog HSS system, virtually all studies of mammalian BER indicate a dominant role for Polβ [41–43]. We confirmed that same dependence for cell-free extracts of HEK293 cells, in which BER of a linear U:A substrate was essentially resistant to aphidicolin, but was blocked when ddTTP replaced the normal nucleotide, with insertion of ddTMP by Polβ aborting the repair process (Fig. 7A). Similar results were obtained for an F:A substrate, although aphidicolin may have produced a small delaying effect for repair to be completed (Fig. 7B).

We attempted to increase the proportion of Polδ and Polε compared to Polβ by synchronizing HEK293 cells in G_1_-S by means of a double-thymidine block (see the “Materials and methods” section). While the procedure resulted in cells that were >95% in G_1_-S (Fig. 7C and D), it did not significantly change the sensitivity of WCE repair to ddTTP for the linear BER substrates (Fig. 7E and F). The BER activity in the synchronized WCE also remained resistant to aphidicolin (Fig. 7E and F).

**Figure 8.**
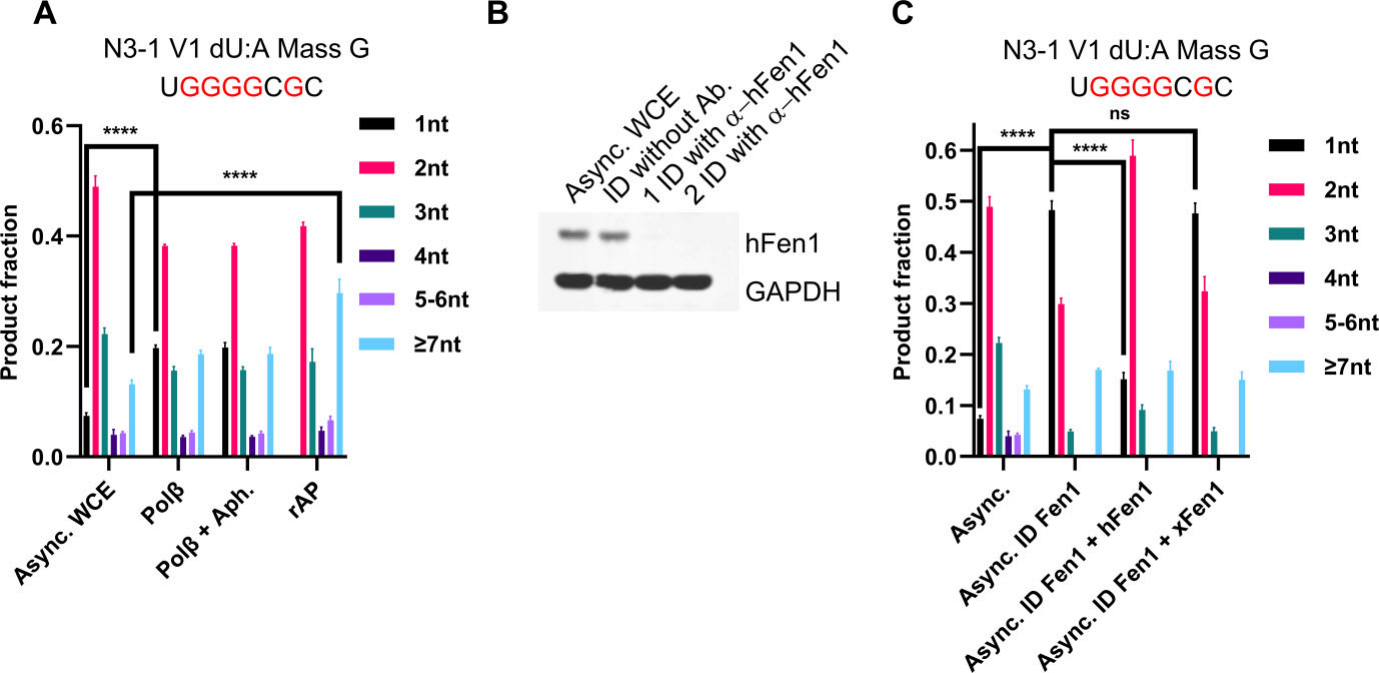
hFen1 immunodepletion increased SP-BER product. (**A**) The BER patch size for a U:A and rAP:A plasmid substrate generated by asynchronous HEK 293 FT WCE or WCE supplemented with Polβ (50 nM) and aphidicolin (100 μg/ml). Aph, aphidicolin. **** indicates *P *< .0001. *n* = 3 independent experiments and the data are means ± SD. Async, asynchronous. (**B**) The hFen1 abundance in asynchronous HEK 293 FT WCE and anti-hFen1 antibody immunodepleted WCE were determined using western blot. ID: Immunodepletion. (**C**) The BER patch size for a U:A plasmid substrate generated by asynchronous HEK 293 FT WCE, hFen1 immunodepleted WCE, hFen1 (150 nM) supplemented hFen1 immunodepleted WCE, and xFen1 (150 nM) supplemented hFen1 immunodepleted WCE. **** indicates *P *< .0001. ns, not significant. *n* = 3 independent experiments and the data are means ± SD.

When the repair patch size assay was applied to the products of HEK293 WCE, the 2-nt product dominated for two different U:A substrates (Fig. 8A). When more Polβ was added to the extract, there was a ∼3-fold increase in the proportion of 1-nt repair products at the expense of the 2-nt and 3-nt products, and the further addition of aphidicolin did not change that result (Fig. 8A). As expected, the rAP substrate produced no detectable 1-nt product, with 2-nt and 3-nt repair patches accounting ~2/3 of the total, and a higher proportion of ≥7-nt patches than seen with U:A under any circumstances (Fig. 8A).

Although we were unable to deplete xFen1 from the *Xenopus* HSS, the removal of the nuclease was essentially complete for the HEK293 WCE (Fig. 8B). For the version 1 U:A substrate, hFen1 depletion produced a dramatic increase in the fraction of 1-nt repair products generated by the WCE of HEK293 (Fig. 8C). Notably, the readdition of hFen1 to the depleted WCE restored the pattern seen with non-depleted extract (Fig. 8C), indicating that no other important proteins were co-depleted by the procedure. In contrast, adding xFen1 to the depleted extract gave no significant difference compared to the depleted WCE (Fig. 8C). That result suggests that interactions of hFen1 with its partners are as important as the level of DNA polymerases in governing the amount of DNA repair synthesis that occurs.

**Figure 9.**
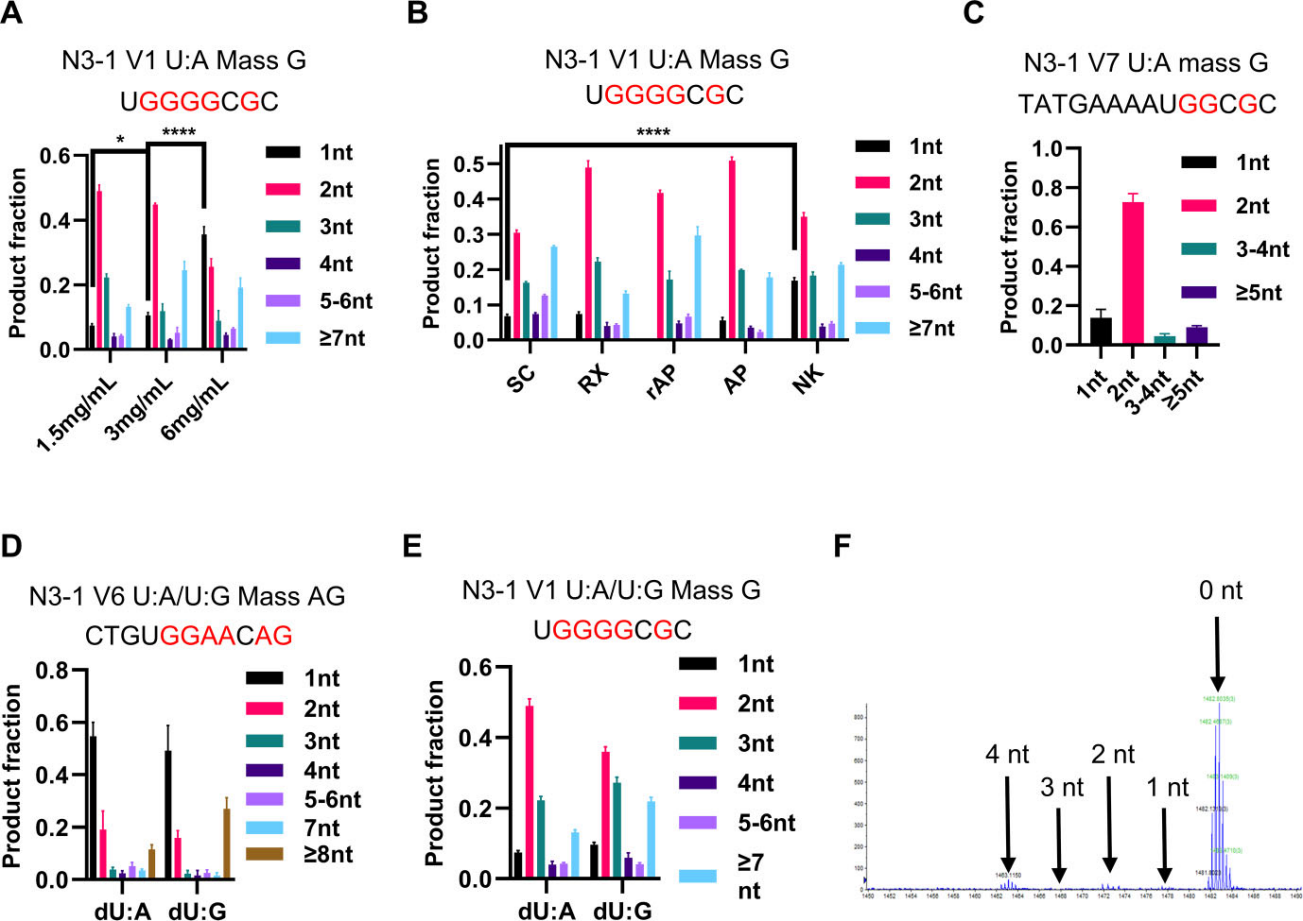
The effect of protein concentration, substrate topological state, and sequence context on BER patch size distribution on HEK 293 WCE. (**A**) The BER patch size generated by the indicated asynchronous HEK 293 FT WCE concentrations is shown for the N3–1 V1 U:A plasmid substrate. (**B**) Various lesions in relaxed or supercoiled N3–1 V1 plasmid substrate. (**C**) The BER patch size generated by asynchronous HEK 293 FT WCE is shown for the N3–1 V7 U:A plasmid substrate (C), N3–1 V6 U:A/U:G plasmid substrate (**D**) and N3–1 V6 U:A/U:G plasmid substrate. (**E**) *n* = 3 independent experiments, and the data are means ± SD. * indicates *P *< .05. **** indicates *P *< .0001. (**F**) A sample mass spectrometry result from asynchronous HEK 293 FT WCE incubated with N3-1 V7 U:A plasmid substrate. 0 nt, 1 nt, 2 nt, 3 nt, and 4 nt indicate the repair products that went through 0 nt, 1 nt, 2 nt, 3 nt, and 4 nt + longer 5′ excision.

The repair patches generated by HEK293 WCE showed a pronounced dependence on the concentration of extract present in the assay. Increasing the concentration by six-fold shifted the pattern to ∼35% 1-nt and ∼25% 2-nt products, with the fraction of ≥7-nt products roughly doubling (Fig. 9A). Possible reasons for this dependence are considered in the “Discussion” section. However, changing the topological state of a U:A substrate generated modest effects, but with the 2-nt products always predominant, ∼30% for a supercoiled substrate, and 40%–50% for the others (Fig. 9B).

Strikingly, changing the sequence around the repair site could dramatically shift the repaired products. Changing from version 7 to the version 6 sequence resulted in ∼50% 1-nt products, which was the case for both U:A and U:G substrates (Fig. 9C and D). In contrast, the version 1 substrate sequence again gave mostly 2-nt (40%–50%) and 3-nt (20%–30%) products for both the U:A and the U:G substrates (Fig. 9D). Unlike the *Xenopus* system, there was no detectable 5′ excision associated with BER in HEK293 WCE (Fig. 9E).

## Discussion

The molecular factors governing the deployment of BER pathways have been unclear ever since LP-BER was discovered [44–46]. Some researchers have proposed that this process is largely governed by the DNA glycosylases operating at the start of conventional BER [47, 48]. However, in our experiments eliminating the DNA glycosylase step by UDG-pretreatment (generating the AP substrate) had little to no effect on the BER patch size (Fig. 4). There is also evidence for the importance of DNA polymerases acting downstream of base excision and AP site cleavage [7, 17, 49]. Indeed, a study of *Xenopus* provided the earliest hint of LP-BER, with the demonstration that PCNA is an essential factor for repair in fractionated egg extracts [50], which suggested the involvement of the DNA replication polymerases δ and ε [50]. Later studies provided conflicting results on this topic, with some suggesting that Polβ in mammalian cell extracts is capable of supporting LP-BER without the participation of other DNA polymerases [25].

In this study, we explored the role of DNA polymerases in *Xenopus* HSS extracts to support the BER of various DNA lesions, in differing sequence contexts, and with varying superhelicity. We found that virtually all of the BER carried out in the HSS depends on the replication polymerases, Polδ and Polε, not only for lesions that require LP-BER, but also for lesions such as uracil, which could readily be handled by SP-BER [35–38]. That result evidently reflects the low level of Polβ detected in *Xenopus* eggs [32], which is consistent with the eggs being poised to conduct immediate DNA replication upon fertilization [51, 52]. There appears to be no other limiting factor, since the addition of recombinant Polβ restored BER even in the presence of the Polδ and Polε inhibitor aphidicolin. While Polα is also inhibited by aphidicolin, a more specific inhibitor of that enzyme (ST1926) had no effect on BER in the HSS system (Supplementary Fig. S8) [53, 54].

In WCE of HEK293 cells, however, BER was largely dependent on Polβ, as judged by its resistance to aphidicolin and sensitivity to ddTTP. Attempting to increase the ratio of Polδ and Polε to Polβ by synchronizing did not change this dependence. On the other hand, immunodepletion of Fen1 from the WCE dramatically increased the fraction of 1-nt products, while the readdition of recombinant hFen1 mostly restored the pattern seen with nondepleted WCE. The addition of the *Xenopus* xFen1 protein did not have that effect, although we confirmed that the protein retains its flap-excision activity. Collectively, these observations indicate that the interactions of other repair proteins with the DNA polymerases are important in promoting the LP-BER and that the *Xenopus* protein may lack the key interactions required for the human system.

The BER products in this system were determined using a much-improved mass-labeling approach (Fig. 3), with “heavy” nucleotides replaced by normal-mass residues during DNA repair synthesis. Subsequent mass spectrometry analysis yields a precise readout of the number of nucleotides replaced during repair. For a U:A lesion, most of the products had exactly 2 nucleotides replaced, which is typically scored as LP-BER, although in most systems a range of longer products is also observed [11, 55]. The addition of Polβ shifted the pattern to predominantly 1-nt (SP-BER) repair patches, and the addition of aphidicolin further suppressed the 2-nt product. Recombinant Polλ gave similar results. Most interestingly, the shift to SP-BER did not depend on the DNA polymerase activity of Polβ: only the 5′-dRp lyase activity was required to support 1-nt BER repair synthesis. When active, these DNA polymerases do participate, as demonstrated by the ability of ddTTP to lead to dead-end intermediates in the presence of Polβ or Polλ (Figs 4B and 6C).

The repair patches determined for a range of BER substrates showed 2-nt products predominating for U:A, AP:A, and rAP:A substrates. For the latter, an LP-BER substrate, there was no 1-nt product detected, but there was also ∼25% of 3-nt and ≥7-nt products. Only prior nicking of the AP site gave a significant increase in the fraction of 1-nt products. Interestingly, changing the downstream sequence to be less G-rich gave rise to longer repair patches for all the substrates (Fig. 5C), which is consistent with easier strand displacement for a less GC-rich sequence, and the result indicates the role played by the surrounding DNA sequence itself. The longer products observed for the precleaved substrate in the lower-GC context are consistent with that conclusion.

The topological state of the probe substrate also had important effects. The circular substrates, as prepared, were in a relaxed state after the *in vitro* manipulations, but negative supercoiling was introduced *in vitro* using DNA gyrase [56]. The supercoiling resulted in a range of longer repair products for both sequence contexts and reduced the 2-nt products to a minority in all cases. It is conceivable that the negative supercoils enable easier DNA unwinding, but the repair intermediates involve DNA breakage, which ought to eliminate that effect. Therefore, it seems much more likely that the supercoiling affects the assembly of proteins [57] prior to the start of BER, perhaps including helicases to promote unwinding. Since DNA in cells is negatively supercoiled, this situation would more accurately reflect *in vivo* BER.

We found significant excision *upstream* of the lesion for all the substrates. The features of such 5′ excision are interesting, in that the U:A substrate generated only 10% repair products with no 5′ excision, with most showing 1-nt or 2-nt upstream excision. Notably, the addition of Polβ, which lacks an intrinsic 3′ to 5′ exonuclease activity [58], increased the 2-nt 5′ excision product dramatically, to ∼70% of the products. After the excision of U, the 2-nt product was predominant for all three substrates (AP:A, rAP:A, and nicked), with 0-nt (no 5′ excision) being the only other significant product in a nearly bimodal distribution. Supercoiling these substrates gave a significant change for only one, U:A, increasing the proportion of 2-nt excision to ≥50%. But whatever process removes upstream DNA during BER, it does not efficiently excise 3′-terminal 2′,3′-dideoxynucleotides: the forced incorporation of these by added Polβ or Polλ generated dead-end repair intermediates, which were not removed even with extended incubation. In contrast to the *Xenopus* system, there was no detectable 5′ excision during BER by WCE of HEK293 cells.

DNA polymerases are central to nearly all DNA repair pathways [59–62]. However, the amounts of the individual enzymes can vary during the cell cycle or upon differentiation [19]. Most studies in mammalian systems have not employed synchronized cells, so the extracts are inevitably a mixture that samples cells from throughout cycle, with the proportion corresponding to S-phase usually being minor. Consequently, such studies would over-report the role of enzymes such as Polβ compared to the contributions of Polδ and Polε. We would expect that the relative capacity of SP- and LP-BER would vary with the cell cycle, along with the ability of cells to deal with lesions that require the latter pathway. In fact, it is possible that there is almost no such thing as SP-BER, since at least 90% of the HSS repair products in relaxed DNA substrates result from the excision of one or more nucleotides upstream (5′) of the lesion site, such that even the 1-nt repair patch products mostly correspond to the replacement of ≥2 nucleotides.

Fortier *et al.* [63] reported that, for zebrafish development, BER switched from an aphidicolin-sensitivity to an aphidicolin-insensitivity pattern during early embryo development. That is consistent with our finding that replicative DNA polymerases are involved in both SP-BER and LP-BER in *Xenopus* egg HSS. They also observed an increase in total BER activity in zebrafish embryo extracts from 0 to 72 h post-fertilization [63]. On the other hand, synchronizing HEK293 cells in G_1_-S, gave no major change in the BER processes, which suggests that cyclic changes in the levels of the replication DNA polymerases are not the major determinant of BER pathways in HEK293. The situation in differentiated cells is skewed more greatly, resulting in LP-BER deficiency [15, 16].

In contrast to the zebrafish study, we were surprised to find no detectable repair of 8-oxoG in the HSS system, given the robust repair of uracil that we demonstrated. Proteomic analysis of *Xenopus* eggs [32] indicates that the level of the OGG1 DNA glycosylase and the 8-oxoG:A-specific MYH glycosylase is indeed very low. These observations seem at odds with the very well-substantiated mutagenic effects of 8-oxoG and the multiple pathways that work to prevent that outcome [64–67]. This question is further honed by the need to limit the occurrence of mutations early during development. One possibility is that the pre-fertilization lifetime of the frog eggs in nature is rather short, limiting the amount of 8-oxoG that might accumulate prior to fertilization. The lower-temperature lifestyle of frogs compared to mammals would further limit the formation of many damages, including 8-oxoG. Consequently, repair pathways such as nucleotide excision may be sufficient to handle the mutagenic threat. What is unclear is how, in the absence of MYH to act on 8-oxoG:A pairs (removing the A inserted during replication of a template containing 8-oxoG), NER or other pathways could act preferentially on the newly synthesized strand. Perhaps mismatch repair has a critical role in the early cell divisions of development. Indeed, the possible post-fertilization changes in *Xenopus* BER are of particular interest. Unfortunately, we were able to obtain only limited material from stage 18 *Xenopus* embryos, but that did not exhibit repair activity for the 8-oxoG:C, U:A, or F:A substrates (Supplementary Fig. S9). It seems most likely that the extraction process led to the loss of these activities. It is also worth noting that the active accumulation of 8-oxoG may influence embryonic development and cell differentiation by influencing epigenetic modifications of chromatin [68].

Our findings provide key insights into the molecular determinants of BER, emphasizing the roles of replication polymerases, DNA topology, and sequence context in shaping repair pathway selection. We employed microinjection to introduce plasmid substrates directly into *Xenopus* eggs to further explore BER in a physiological setting. Remarkably, the injected plasmid substrates were fully repaired within 15 min (Supplementary Fig. S10), underscoring the potential of our engineered plasmids as novel tools for studying *in vivo* BER reactions. Notably, even a modest two-fold change in *Xenopus* HSS concentration significantly altered the BER patch size distribution pattern (Supplementary Fig. S11), suggesting that *in vivo* BER could differ dramatically from the *in vitro* BER, with an even stronger bias toward LP-BER. However, technical limitations in isolating sufficient high-purity plasmids prevented mass spectrometry analysis of *in vivo* repair patches. Future work will aim to overcome these challenges, enabling a deeper understanding of BER efficiency and fidelity during early embryogenesis.

## Supplementary Material

gkaf1326_Supplemental_File

## Data Availability

The data underlying this article are available in the article and in its online supplementary data.
