# Supplementary material for: Pathways and products of base excision DNA repair in Xenopus laevis eggs: contrast with human cell pathways
Source: Nucleic Acids Res. 2025 Dec 10;53(22):gkaf1326. doi: 10.1093/nar/gkaf1326 (PMC12693518; doi:10.1093/nar/gkaf1326)
Supplement: gkaf1326_Supplemental_File [file gkaf1326_supplemental_file.pdf]

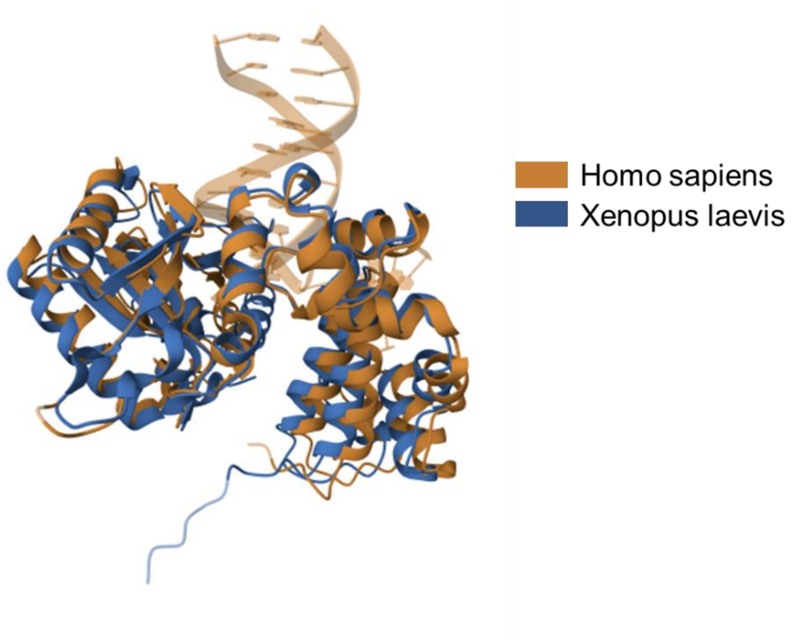

RMSD (root mean square deviation)	2.83
TM-Score (template modeling score)	0.87
Identity	83%
Aligned Residues	328
Length	334
Modeled Residues	334

Supplementary Figure S1. Pairwise structural alignment of Homo sapiens DNA polymerase β (PDB: 1BPX) and *Xenopus* laevis DNA polymerase β (AlphaFold prediction: O57383) reveals a high degree of conservation between these two species.

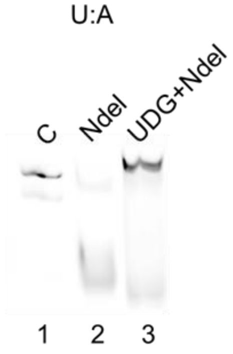

Supplementary Figure S2. NdeI is active on a recognition sequence containing U but is blocked when converted to an AP site. Full-length substrate (indicated by C above lane 1), NdeI-digested U:A substrate (lane 2), and an AP:A substrate incubated with NdeI (lane 3) were resolved by denaturing polyacrylamide gel electrophoresis and imaged using a fluorescent imager.

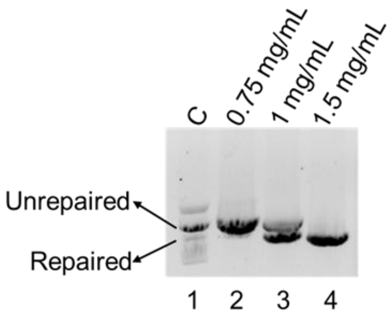

Supplementary Figure S3. Plasmid substrate repair as a function of *Xenopus* HSS concentration. An ethidium bromide-containing agarose gel image showing the repaired product of plasmid substrate by *Xenopus* incubated for 60 min with HSS at different concentrations, with the repaired substrate susceptible to UDG and NdeI cleavage (lanes 2-4). Plasmid substrate was digested with UDG and NdeI and loaded in lane 1 to serve as a marker.

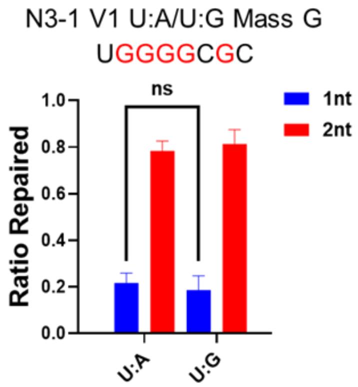

Supplementary Figure S4. The base paired with U did not affect the BER patch size. The N3-1 V1 probe was used to determine the BER patch size generated by *Xenopus* HSS with U:A or U:G plasmid substrates. There were no detectable repair patches >2 nt. Comparisons were made via two-tailed student’s t-test. *n* = 3 independent experiments and the data are means ± SD.

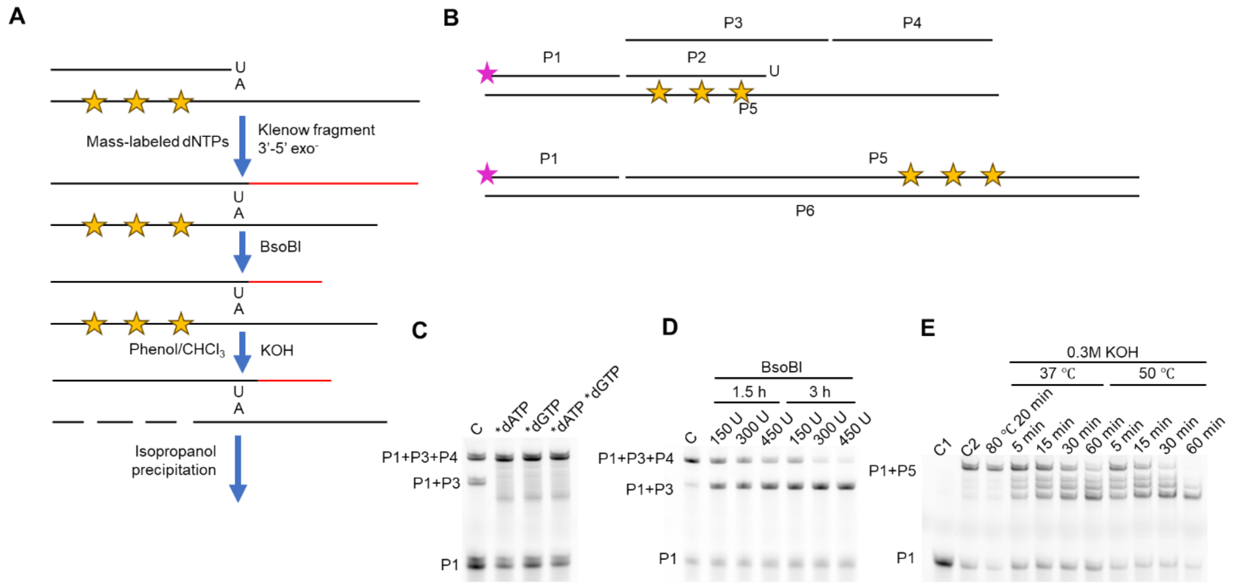

Supplementary Figure S5. Enzymatic method for synthesizing isotopically-labeled DNA oligonucleotides. **A**, Overview of the oligo swapping procedure (see Methods for details). **B**, Diagram of oligonucleotides used in assay development experiments shown in **C**, **D**, and **E**. Magenta stars indicate TAMARA labels, while the yellow stars indicate ribonucleotide residues. **C**, Primer extension from a 3′-terminal dUMP residue with isotopically-labeled dNTPs. P1, P2, and P5 were annealed and ligated by T4 DNA ligase. The 3′-exonuclease-deficient Klenow fragment was used to extend the primer with normal dNTPs or mass-labeled [^{13}C, ^{15}N]dATP (lane2), [^{13}C, ^{15}N]dGTP (lane 3) or both (lane 4). Reaction products (lanes 2-4) and markers were electrophoresed as Fig. S2. The faint band is due to small amounts of ligated P1+P2. Note that an excess of the P1 oligonucleotide was present. **D**, BsoBI activity assay. The P1, P3, P4, and P5 oligonucleotides were annealed and ligated using T4 DNA ligase. The resulting products were incubated with increasing amounts of BsoBI for the indicated times. The digestion products were electrophoresed as Fig. S1. **E**, Alkaline-induced cleavage of embedded ribonucleotide residues. P1, P5, and P6 were annealed and ligated using T4 DNA ligase. The resulting product was incubated with 0.3 M KOH at the indicated times and temperatures and then electrophoresed as in Fig. S1.

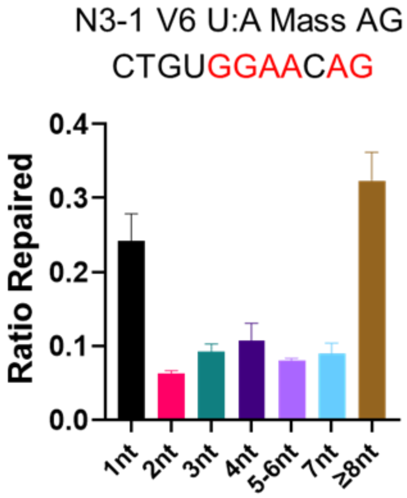

Supplementary Figure S6. Reduced downstream GC content in the substrate leads to longer BER patch size. *n* = 3 independent experiments and the data are means ± SD.

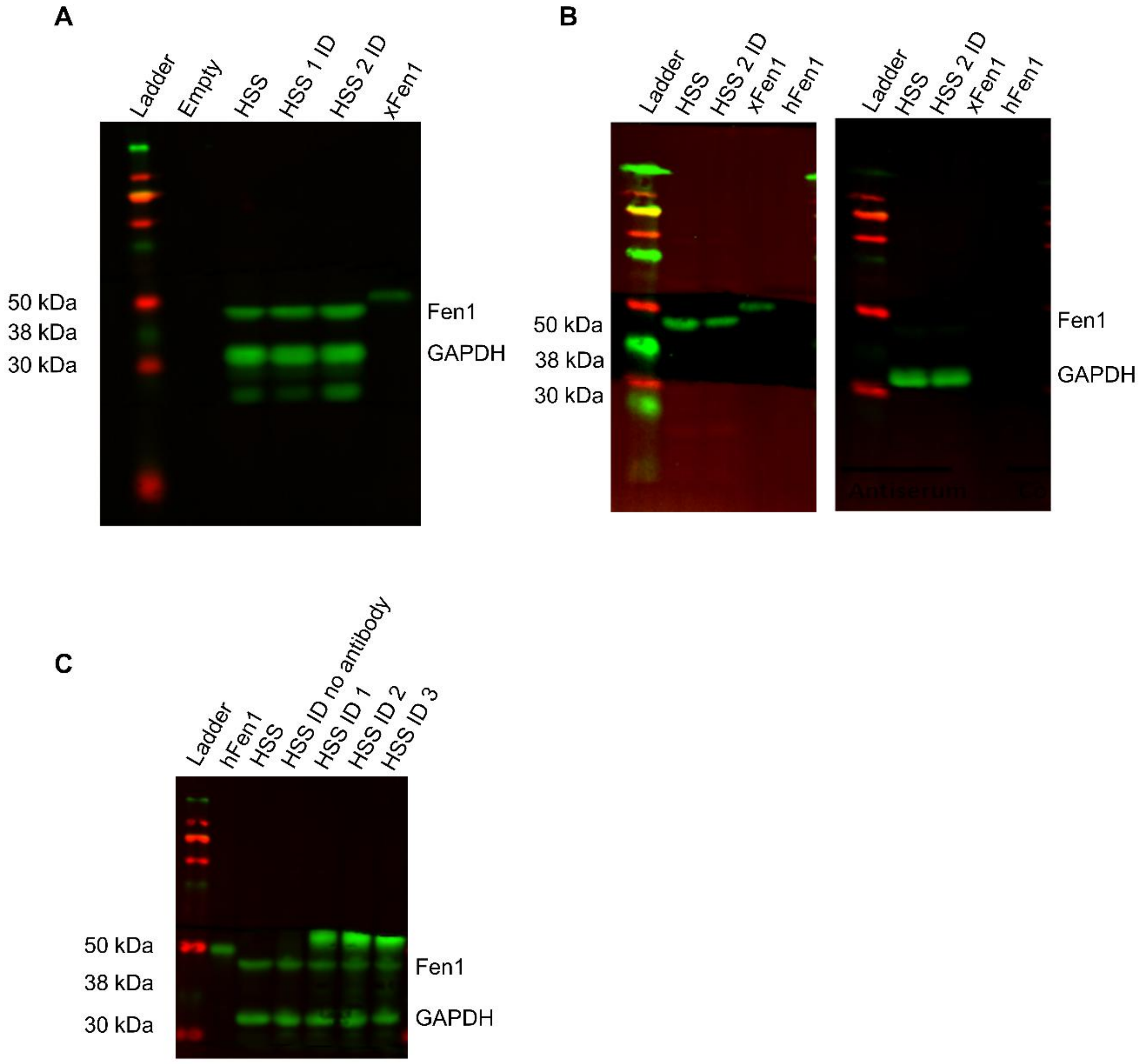

Supplementary Figure S7. Attempted immunodepletion of xFen1 from HSS. **A**. ID of xFen1 in 50 µL HSS using 100 µL anti-xFen1 antiserum and 50 µL protein A magnetic beads each round for 2 rounds. **B**. ID of xFen1 in 50 µL HSS using 25 µL anti-hFen1 antibody and 50 µL protein A magnetic beads each round for 2 rounds. **C**. ID of xFen1 in 50 µL HSS using 100 µL anti-xFen1 antiserum and 100 µL protein A sepharose each round for 3 rounds. ID: Immunodepletion.

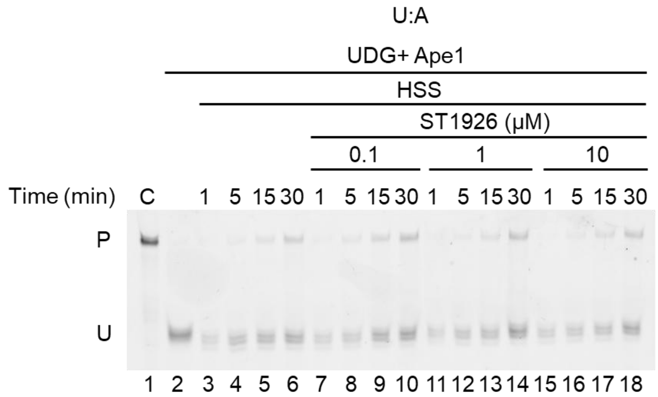

Supplementary Figure S8. Polα inhibition did not slow down the BER rate. HSS was pre-incubated with Polα inhibitor ST1926, the U:A substrate was added, and incubation was carried out for the indicated time. UDG +Ape1 was used to nick the unrepaired portion. Full-length substrate (indicated by C above lane 1), UDG + Ape1 digested substrate (lane 2), and UDG + Ape1 digested, HSS-repaired samples (lanes 3–6) were electrophoresed in a denaturing polyacrylamide gel, then imaged using a fluorescent imager.

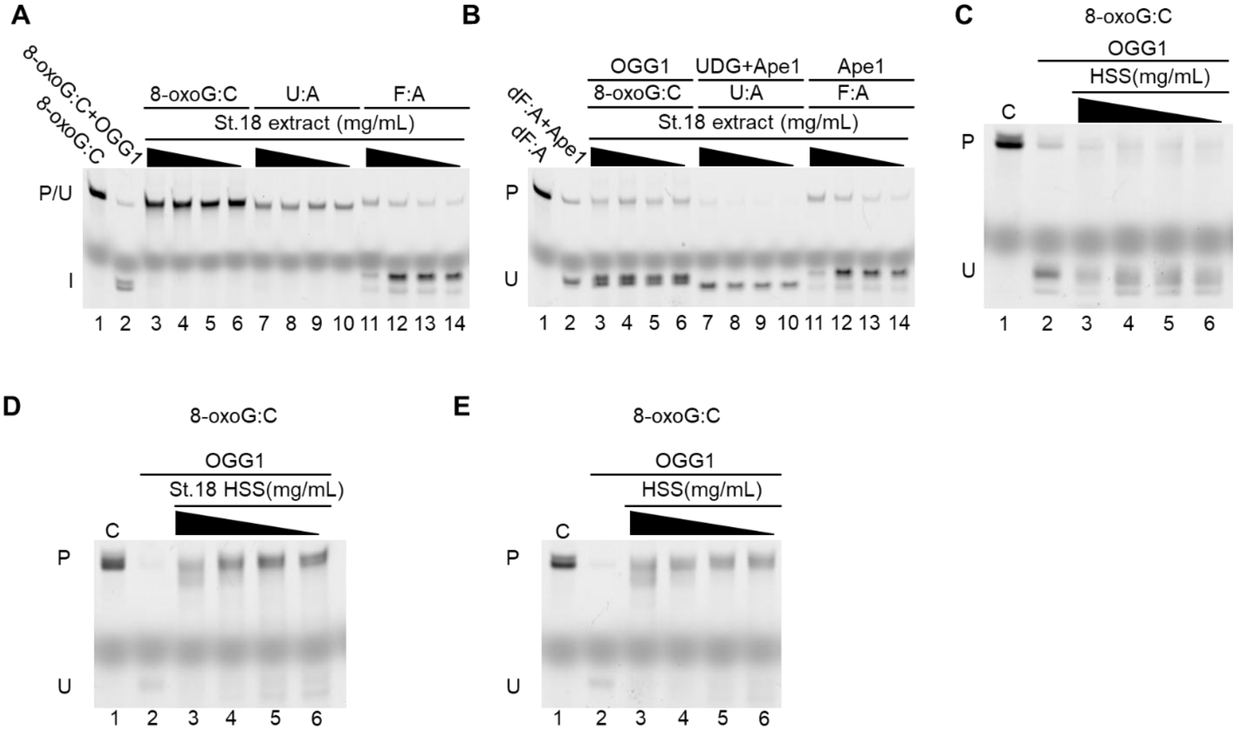

Supplementary Figure S9. Very low BER activity in *Xenopus* stage 18 embryo extract and lack of 8-oxoG repair in both stage 18 extract and HSS. **A**, Increasing concentrations of *Xenopus* embryo extract were incubated with 8-oxoG:C (lanes 3-6), U:A (lanes 7-10), and F:A (lanes 11-14) substrates for 60 min. The products were electrophoresed and imaged as for Fig. S1. The concentrations used were 3 mg/mL, 1.5 mg/mL, 0.75 mg/mL, and 0.6 mg/mL. **B**, Lanes 3-14 in A were digested with indicated enzymes to nick the unrepaired portions and loaded in lanes 13-14. **C**, Lack of 8-oxoG:C repair by *Xenopus* HSS. **D** and **E**, Exogenous Ogg1 supported 8-oxoG BER in *Xenopus* extracts. Stage 18 embryo extract (**D**) or *Xenopus* HSS (**E**) were supplemented with recombinant Ogg1 and incubated with 8-oxoG:C substrate. P the repaired product, and U the unrepaired substrate or a repair intermediate.

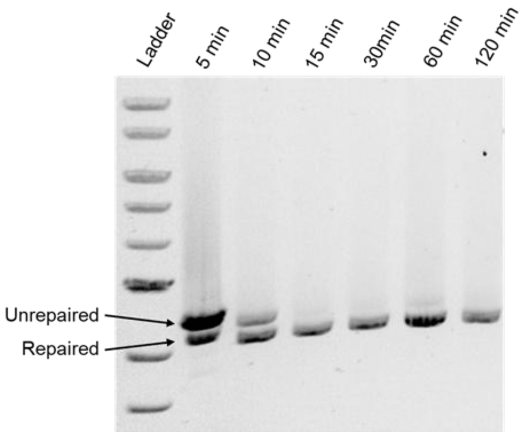

Supplementary Figure S10. Intact *Xenopus* eggs have robust BER activity. The isotopically-labeled N3-1 U:A plasmid substrate was microinjected into *Xenopus* eggs (50 for each time point) and recovered at the indicated times. The recovered DNA was digested with UDG and NdeI to identify repaired molecules. The resulting products were electrophoresed on an ethidium bromide-containing agarose gel.

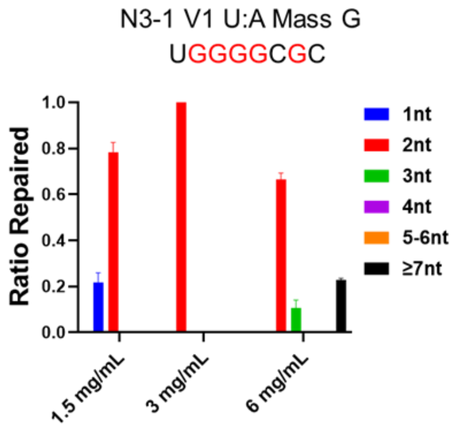

Supplementary Figure S11. Effect of *Xenopus* HSS protein concentration on BER patch size. The BER patch size generated by the indicated HSS concentrations is shown for the N3-1 V1 U:A plasmid substrate. Note that repair patches of 4 nt and 5-6 nt were not detectable. n = 3 independent experiments and the data are means ± SD.

Supplementary Table

Name	Sequence
ssN3-1 V1 X:A	5′-phos CAAGCTTGCATGCCTGCAGGTCGACTTCGCGAGCGGCTGAGGTGCTCTTCACATATGGGGCGCTGAGGACGTCGGGATCCCCGGGTACCGAGCTCGAAT
ssN3-1 V1 X:G	5′-phos CAAGCTTGCATGCCTGCAGGTCGACTTCGCGAGCGGCTGAGGTGCTCTTCACATACGGGGCGCTGAGGACGTCGGGATCCCCGGGTACCGAGCTCGAAT
ssN3-1 V6 X:A	5′-phos CAAGCTTGCATGCCTGCAGGTCGACTTCGCGAGCGGCTGAGGTGCTCTTCAGCTGTGGAACAGCTGAGGACGTCGGGATCCCCGGGTACCGAGCTCGAAT
ssN3-1 V7 X:A	5′-phos CAAGCTTGCATGCCTGCAGGTCGACTTCGCGAGCGGCTGAGGTGCTCTTCACATATGAAAATGGCGCTGAGGACGTCGGGATCCCCGGGTACCGAGCTCGAAT
NdeI U FWD	5′-GCGAGCGGGCTGAGGGCTCTTCACATA/U/GGGGCGCTGAGGACGTCGCCGCT/TAMARA/
NdeI U REV	5′-AGCGGCGACGTCCTCAGCGCCCCATATGTGAAGAGCCCTCAGCCCGCTCGC
51U FWD	5′-/TAMARA/TTGCATGCCTGCAGGTCGA/U/TCTAGAGGATCCCCGGGTACCGAGCTCGA
51F FWD	5′-TGCTTGCATGCCTGCAGGTCGA/F/TCTAGAGGATCCCCGGGTACCGAGCTCGA/TAMARA/
518-oxoG FWD	5′-/TAMARA/TTGCATGCCTGCAGGTCGA/8-oxoG/TCTAGAGGATCCCCGGGTACCGAGCTCGA
51A REV	5′-TCGAGCTCGGTACCCGGGGATCCTCTAGAATCGACCTGCAGGCATGCAAGCA
51C REV	5′-TCGAGCTCGGTACCCGGGGATCCTCTAGACTCGACCTGCAGGCATGCAAGCA
51G REV	5′-TCGAGCTCGGTACCCGGGGATCCTCTAGAGTCGACCTGCAGGCATGCAAGCA
N3-1 U iso G	5′-phos TGAGG/bioT/GCTCTTCACATA/U/*G*G*G*GC*GC
UP20-C20 (P1)	5′-/TAMARA/ phos CGGTGATATGCAGTCAGTAC
Ver 6 3′U (P2)	5′-phos TGAGGTGCTCTTCAGCTG/U/
Ver 6 U Fl(P3)	5′-phos TGAGGTGCTCTTCAGCTGTGGAACAGC

Ver 6 mass G syn 3′ (P4)	5′-phos TCGAGACGTCGGGA
Ver 6 test comp (P5)	5′-phos TCCCGACGTCTCGAGCTGTTCCACAG/rC/TGAA/rG/AGCA/rC/CTCAGTACTGACTGCATATCACCG
RNA/U lysis test (P6)	5′-TTGTATGAGGTGCTCTTCAGCTGTGGAACAGCTCGAGACGTCGTACTGACTGCATATCACCG
Ver 6 3′U	5′-phos TGAGGTGCTCTTCAGCTGU
Ver 6 mass AG syn comp R	5′-TCCCGACGTCTCGAGCTGTTCCACAG/rC/TGAA/rG/AGCA/rC/CTCATACAA
Ver 7 3′ G	5′-phos TGAGGTGCTCTTCACATATG
Ver 7 mass AG syn comp (R)	5′-TCCCGTCGTCTCGGGCGCCATTTTCATAT/rG/TGAA/rG/AGCAC/rC/TCATACAA

Supplementary Table 1: Sequences of the oligonucleotides used in this study. Modifications are indicated in red, with rC and rG indicating ribonucleotides, and *G indicating [^{13}C, ^{15}N]dGMP (mass-labeled) residues. The N3-1 U iso G oligonucleotides was synthesized by the W.M. Keck Oligonucleotide Synthesis Facility, Yale University School of Medicine, New Haven, CT. All other oligonucleotides were synthesized by Integrated DNA Technologies.
